# A Belgian Population-Based Study Reveals Subgroups of Right-sided Colorectal Cancer with a Better Prognosis Compared to Left-sided Cancer

**DOI:** 10.1093/oncolo/oyad074

**Published:** 2023-04-18

**Authors:** Katleen Janssens, Erik Fransen, Guy Van Camp, Hans Prenen, Ken Op de Beeck, Nancy Van Damme, Marc Peeters

**Affiliations:** Center of Medical Genetics, University of Antwerp and Antwerp University Hospital, Prins Boudewijnlaan 43, Edegem, Belgium; Center of Oncological Research (CORE), University of Antwerp, Universiteitsplein 1, Wilrijk, Belgium; Center of Medical Genetics, University of Antwerp and Antwerp University Hospital, Prins Boudewijnlaan 43, Edegem, Belgium; StatUa Center for Statistics, University of Antwerp, Antwerp, Belgium; Center of Medical Genetics, University of Antwerp and Antwerp University Hospital, Prins Boudewijnlaan 43, Edegem, Belgium; Center of Oncological Research (CORE), University of Antwerp, Universiteitsplein 1, Wilrijk, Belgium; Center of Oncological Research (CORE), University of Antwerp, Universiteitsplein 1, Wilrijk, Belgium; Department of Oncology, Antwerp University Hospital, Wilrijkstraat 10, Edegem, Belgium; Center of Medical Genetics, University of Antwerp and Antwerp University Hospital, Prins Boudewijnlaan 43, Edegem, Belgium; Center of Oncological Research (CORE), University of Antwerp, Universiteitsplein 1, Wilrijk, Belgium; Belgian Cancer Registry, Koningsstraat 215, Brussels, Belgium; Center of Oncological Research (CORE), University of Antwerp, Universiteitsplein 1, Wilrijk, Belgium; Department of Oncology, Antwerp University Hospital, Wilrijkstraat 10, Edegem, Belgium

**Keywords:** colorectal neoplasms, tumor biomarkers, survival, population register, primary tumor location

## Abstract

**Background:**

Patients with left-sided colorectal cancer (L-CRC) are known to have a significantly better prognosis than those with right-sided CRC (R-CRC). It has been hypothesized that *RAS, BRAF* mutations, or deficient mismatch repair status (MMR) might be responsible for the prognostic effect of primary tumor location (PTL). This study aims to evaluate the prognostic effect of PTL in the Belgian population and to determine the role of biomarkers (MMR, *BRAF,* and *RAS* status) in this effect.

**Patients and Methods:**

We performed a retrospective analysis of Belgian Cancer Registry data. First, we studied the prognostic effect of PTL on 5-year relative survival of 91,946 patients diagnosed with CRC (all stages) from 2004-2015. Second, we investigated the interaction between biomarkers and the prognostic effect of PTL in 1818 patients diagnosed with stage IV CRC in 2014-2015.

**Results:**

L-CRC was associated with a significantly better 5-year relative survival compared to R-CRC in all stages and ages combined (68.4%, 95% CI, 67.7-69.1% vs 65.6%, 95% CI, 64.7-66.4%). Also, when stratified by age, sex, and stage, the prognosis of L-CRC was better compared to R-CRC in most subgroups. Only in stage II and certain subgroups of elderly patients, the opposite was observed. Furthermore, our data showed that none of the biomarkers had a significant interaction with the effect of PTL on survival.

**Conclusion:**

This population-based study confirms that L-CRC is associated with significantly better relative survival compared to R-CRC, in all stages and ages combined. Furthermore, in stage IV L-CRC is associated with a longer survival than R-CRC, regardless of MMR, *RAS,* and *BRAF* status.

## Teaser

Primary tumor location in colorectal cancer (CRC) has been intensively studied, demonstrating different disease characteristics in left- and right-sided CRC. This article evaluates the prognostic effect of primary tumor location in the Belgian population to determine the role of biomarker status (MMR, BRAF, and RAS mutational status).

**Implications for Practice**

Right- and left-sided colorectal cancer (CRC) are 2 subtypes of CRC with distinct clinical and molecular features. This population-based study confirms that relative survival is significantly longer in patients with left-sided CRC compared to right-sided CRC, across all stages and ages combined. Furthermore, in metastatic CRC left-sided tumors are associated with a better survival than right-sided tumors, regardless of RAS and BRAF mutational or mismatch repair status. The complex underlying mechanisms responsible for the difference in prognosis should be identified to gain insight into CRC biology and to allow selection of the optimal therapy for each CRC subtype.

## Introduction

Colorectal cancer (CRC) is the third most frequently diagnosed cancer worldwide and is ranked second in terms of mortality.^1^ During the past years, the impact of primary tumor location in CRC has been intensively studied, demonstrating different disease characteristics in left- and right-sided CRC.^2^

The division in the right and left part of the colon is based on its embryological origin. The right part of the colon originates from the midgut, while the left part is derived from the hindgut. The embryological border between both parts of the colon is located at the proximal two-thirds of the transverse colon.^3^ However, most researchers use the splenic flexure as the boundary between left- and right-sided tumors. In some studies, tumors of the transverse colon are excluded since classifying them as right-sided is not entirely correct.^4^ Furthermore, some authors classify rectal cancers as a third separate group and therefore exclude rectal cancers from left-sided CRC.^5^

From an anatomical perspective, blood supplies, innervations, lymphatic drainages, and lumen environments are among the differences between the right and left colon. Bufill et al (1990) were the first to describe CRC based on the anatomical site, in an attempt to sub-classify CRC.^6^ Right-sided CRC is associated with female gender, old age, *BRAF* mutations, and microsatellite instability, while left-sided CRC is associated with male gender, younger patients, and chromosomal instability. Left-sided tumors often cause infiltrating, constricting lesions, causing obstruction, and early symptoms. In contrast, right-sided tumors are mostly exophytic, polypoid lesions, causing anemia, and unfortunately, symptoms only in later stages.

In recent years, various studies have convincingly shown that patients with tumors originating on the left side of the colon have a significantly better prognosis than those with tumors originating on the right side, in all stages.^7-16^ This means that primary tumor location has a prognostic value as it provides information about the overall cancer outcome, independent of treatment received. However, these conclusions are mostly based on retrospective analysis of data from clinical trials and therefore based on data from selected patients, often in small subgroups. Therefore, these findings need to be confirmed in population-based studies, representing an unbiased set of patients.

Furthermore, the underlying mechanisms that cause this difference in survival have not been identified yet. It has been hypothesized that *KRAS, NRAS,* or *BRAF* mutations and deficient mismatch repair status (MMR) might be partially responsible for the prognostic effect of primary tumor location.

The aim of this study is to evaluate the prognostic effect of primary tumor location in the Belgian population and to determine the role of biomarker status (MMR, *BRAF,* and *RAS* mutational status) in this prognostic effect.

## Patients and Methods

### Database and Study Population

In Belgium, data on patient and tumor characteristics of all newly diagnosed cancers are collected in a national cancer registry; the Belgian Cancer Registry (BCR). In this study, a retrospective analysis of data of the BCR was performed. Data collected and followed by the BCR includes demographic characteristics, primary tumor location, stage at diagnosis, and survival.

All Belgian patients diagnosed with colorectal adenocarcinoma (International Classification of Diseases, Oncology Third Revision (ICD-O-3) histology codes: 8140, 8141, 8210, 8211, 8221, 8260, 8261, 8262, 8263, 8480, 8481, and 8490) were included in this study. All stages (except stage 0, in situ tumors) were included. Patients with tumors located in the appendix (topographical code ICD-O-3: 18.1) and unspecified or overlapping lesions (topographical code ICD-O-3: C18.8, C18.9) were excluded. Patients who died at the time of diagnosis or were diagnosed at autopsy were also excluded.

The anatomical location of tumors was classified according to the Ninth Revision of the International Classification of Diseases (ICD-9). Cancers were classified as left-sided if they were located at the splenic flexure, descending colon, sigmoid, and rectosigmoid. This corresponds to the topographical code ICD-O-3: C18.5, C18.6, C18.7, and C19.9. Right-sided colon cancer was defined as cancer of the caecum, ascending colon, hepatic flexure, and transverse colon, corresponding to topographical code ICD-O-3: C18.0, C18.2, C18.3, C18.4. Rectal cancer was interpreted as a third separate group (topographical code ICD-O-3: C20.9).

The stage at diagnosis was obtained from a compilation of pathological (pTNM) and clinical (cTNM) stages. If both pStage and cStage are available, pStage was used for the combined stage. An exception to this rule was cases with clinical metastases (cM = 1): in this case, the combined stage was IV. If either the pathological or the clinical stage only was available, the combined stage was derived from the available stage. If both pStage and cStage were absent, the combined stage was considered unknown.

In the first analysis, the prognostic effect of primary tumor location (PTL) was studied in all patients diagnosed with CRC (all stages) between January 1, 2004 and December 31, 2015. The last date for follow-up was July 1, 2017. Five-year relative survival was selected as outcome, to correct for competing causes of death. The 5-year relative survival rate was combined with the number of patients at risk in each category (according to age, sex, stage, and primary tumor location) to calculate the number of patients surviving and not-surviving. These numbers were used for further statistical analyses.

In a second analysis, the effect of biomarkers on survival and on the prognostic effect of PTL was investigated in a random sample of approximately 2000 patients diagnosed with de novo stage IV CRC in 2014 and 2015. The data entry was closed in July 2017. The same inclusion and exclusion criteria as the first analysis were applied for the second analysis, but only stage IV CRC was studied. To avoid survival being influenced by a second malignancy, we excluded patients who were diagnosed with a second tumor. However, in situ tumors (stage 0) or basal cell carcinomas were allowed.

We reviewed the histopathological reports of all patients included in the second analysis and collected information about mismatch repair status (deficient mismatch repair status (dMMR) vs. proficient mismatch repair status (pMMR)) and *BRAF, KRAS,* and *NRAS* mutational status (mutant vs. wild-type). Biomarker data was obtained according to the protocols of the laboratory of the treating hospital. This implies that which laboratory techniques were used, which exons of *KRAS* and *NRAS* were assessed and whether mismatch repair testing was done by immunohistochemistry or molecular testing, was decided by the laboratory of the treating hospital.

### Statistical Analyses

Statistical analysis was performed by the statistical software package R version 3.3.2. A *P*-value of < .05 was considered statistically significant.

For the first analysis, we constructed a logistic regression model using the fraction of deceased patients as the dependent variable and location, stage, age, and gender as the independent variables. Across all logistic regression models, 5-year relative survival was entered as outcome variable.

In the second analysis, Cox proportional hazard models were constructed to determine the effect of *KRAS, NRAS, BRAF* mutational status, and MMR status on survival. Overall survival was defined as the time from diagnosis of CRC to death from any cause. Patients who were either (1) alive at the data entry closure date or (2) were lost to ­follow-up were censored at the last date that they were known to be alive.

## Results

### Prognostic Effect of Primary Tumor Location

In the first analysis, the prognostic effect of PTL was studied in patients with CRC diagnosed in 2004-2015. This study population included 91,946 patients: 28,359 (30.8%) with right-sided CRC and 63,587 (69.2%) with left-sided or rectal CRC. Table 1 presents the demographic and clinical characteristics of this study population.

**Table 1.** Demographic and clinical characteristics of patients diagnosed with CRC in 2004-2015.

	Right-sided (*n* = 28,359)	Left-sided (including rectal) (*n* = 63,587)	Total number (*n* = 91,946)
Age			
0-59	3,278 (11.6%)	12,289 (19.3%)	15,567
60-69	5,813 (20.5%)	16,696 (26.3%)	22,509
70-79	9,584 (33.8%)	20,377 (32.0%)	29,961
80+	9,684 (34.1%)	14,225 (22.4%)	23,909
Sex			
Male	13,581 (47.9%)	37,601 (59.1%)	51,182
Female	14,778 (52.1%)	25,986 (40.9%)	40,764
Stage			
I	3,884 (13.7%)	13,886 (21.8%)	17,770
II	9,767 (34.4%)	16,049 (25.2%)	25,816
III	7,880 (27.8%)	16,383 (25.8%)	24,263
IV	4,965 (17.5%)	10,957 (17.2%)	15,922
Unknown	1,863 (6.6%)	6,312 (9.9%)	8,175

Please note that only the first diagnosis of colorectal cancer per individual is included in the survival analysis.

Overall, the 5-year relative survival rate for patients with right-sided CRC was 65.6% (95% CI, 64.7%-66.4%). This is significantly lower compared to 67.5% (95% CI, 66.9%-68.0%) for patients with left-sided and rectal CRC, in all stages combined.

**Figure 1.**
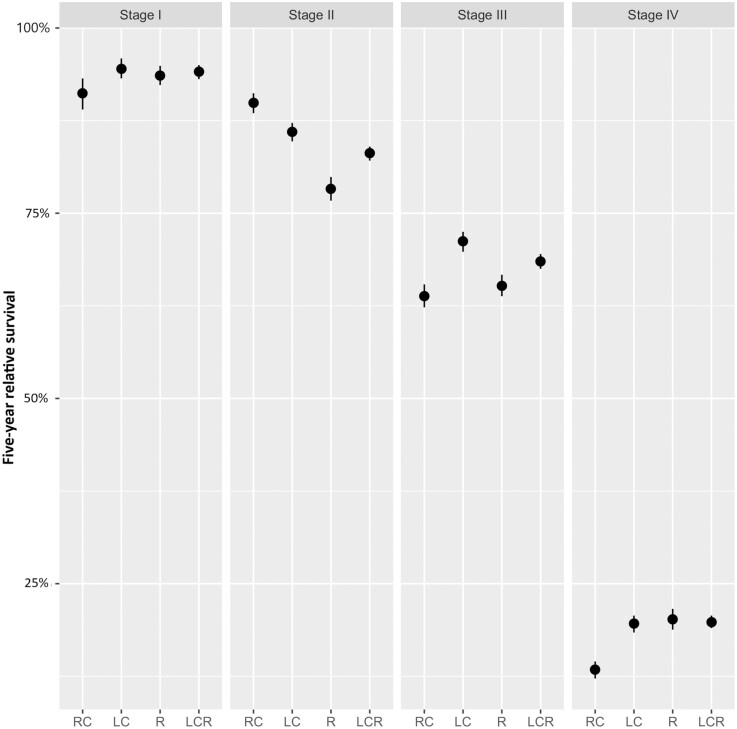
Graphical representation of 5-year relative survival according to location and stage. Left-sided CRC was associated with a significantly better survival in stages I, III, and IV compared to right-sided CRC. Abbreviations: RC, right colon; LC, left colon; R, rectum; LCR, left colon and rectum.

When stratified by stage, left-sided CRC (including rectal cancer) was associated with a significantly better survival in stages I, III, and IV compared to right-sided CRC (Table 2; Fig. 1). Patients with right-sided CRC in these stages had approximately 1.5 times higher odds compared to patients with left-sided CRC (stage I, odds ratio (OR) 1.49; stage III, OR 1.21; and stage IV, OR 1.58). The odds ratio was here defined as the odds to die of cancer during 5-year follow-up in patients with right-sided CRC versus patients with left-sided CRC. However, in stage II we observed the opposite association; right-sided CRC was associated with a significantly better survival compared to left-sided CRC (OR 0.51). When comparing right-sided colon cancer to left-sided colon cancer without rectal cancer, we observed similar results (Supplementary Tables S1 and S2).

**Table 2.** Five-year relative survival rate according to location and stage.

	Stage (combined clinical and pathological)	Number at risk	Five years relative survival (%)	Five years relative survival (95% CI)
Colorectal (all locations combined)	All stages	91,946	66.3	[65.9%:66.7%]
	I	17,961	93.4	[92.5%:94.2%]
	II	26,258	85.4	[84.6%:86.2%]
	III	24,464	66.7	[65.9%:67.5%]
	IV	16,561	17.7	[17.0%:18.4%]
	Unknown	9432	48.8	[47.5%:50.0%]
Right colon	All stages	27,863	65.6	[64.7%:66.4%]
	I	3850	91.2	[89.0%:93.2%]
	II	9699	89.9	[88.5%:91.2%]
	III	7805	63.8	[62.3%:65.4%]
	IV	4938	13.4	[12.2%:14.5%]
	Unknown	1813	37.0	[34.1%:40.0%]
Left colon and rectum	All stages	62,728	67.5	[66.9%:68.0%]
	I	13,784	94.1	[93.1%:95.0%]
	II	15,963	83.1	[82.1%:84.0%]
	III	16,265	68.5	[67.5%:69.5%]
	IV	10,901	19.8	[19.0%:20.7%]
	Unknown	6192	52.0	[50.4%:53.6%]
Left colon	All stages	35,815	68.4	[67.7%:69.0%]
	I	7134	94.5	[93.2%:95.9%]
	II	10,068	86.0	[84.7%:87.2%]
	III	9091	71.2	[69.8%:72.5%]
	IV	6534	19.6	[18.4%:20.7%]
	Unknown	3106	51.4	[49.1%:53.7%]
Rectum	All stages	27,359	66.1	[65.4%:66.9%]
	I	6718	93.6	[92.3%:94.9%]
	II	5918	78.3	[76.7%:79.9%]
	III	7222	65.2	[63.8%:66.7%]
	IV	4396	20.2	[18.8%:21.6%]
	Unknown	3107	52.3	[50.0%:54.5%]

Please note that the total number of patients does not always add up to 91,946 because only the first diagnosis of colorectal cancer per individual is included in the survival analysis. However, when analyzing data per stage, per age, or per year of diagnosis, all colorectal cancers are included (ie, including the following primary colorectal cancers a person develops).

**Figure 2.**
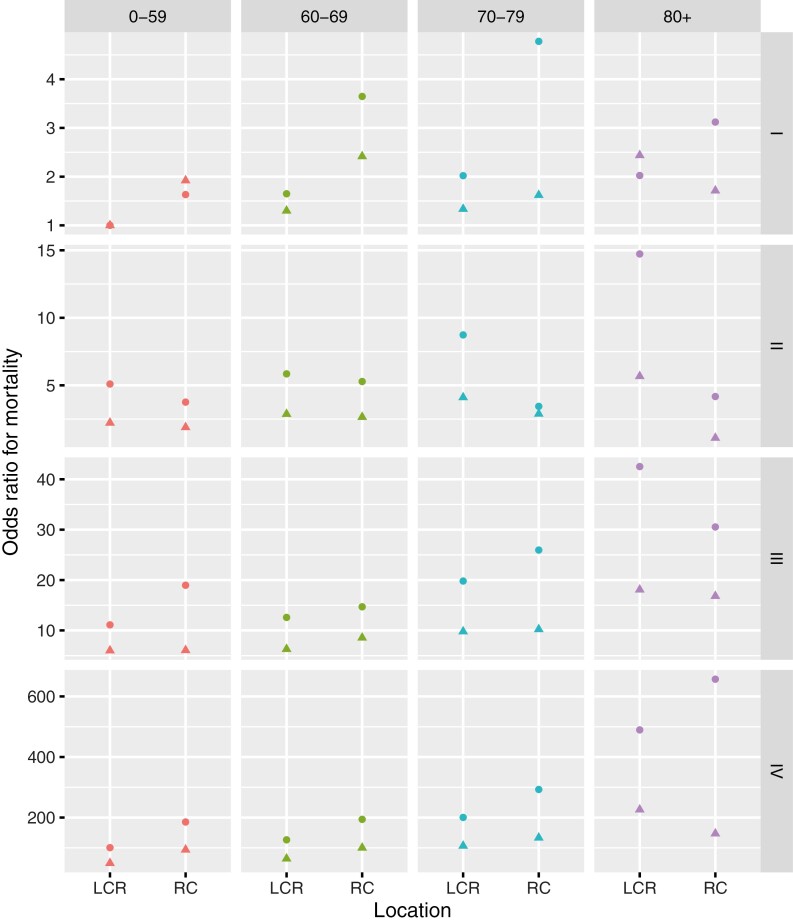
Odds ratios comparing right- to left-sided CRC according to age, stage, and sex. The odds ratio was defined as the odds to die of colorectal cancer during the 5 years follow-up in the non-reference category (right-sided CRC) versus the reference category (left-sided CRC). Five-year relative mortality in stage I, patients with left-sided colorectal cancer aged 0-59 year was used as reference survival (in each sex separately). In most subgroups, left-sided CRC is associated with a significantly better 5-year relative survival compared to right-sided CRC. Only in the subgroups of stage I ­>80-year-old males, stage II >70-year-old males and females, stage III >80-year-old females, and stage IV >80-year-old males, a significantly better survival in right-sided CRC was observed. Dots, females; triangles, males. Abbreviations: RC, right colon; LCR, left colon and rectum.

When analyzing the data stratified by stage, age, and sex, left-sided and rectal CRC was again associated with a significantly better 5-year relative survival compared to right-sided CRC in most subgroups. Only in stage II and certain subgroups of patients of 80-years-old or older (stage I >80­-year-old males, stage III >80-year-old males and females, and stage IV >80-year old males) we observed a significantly longer survival in right-sided CRC. A summary of the odds ratios for mortality comparing right- to left-sided (incl. rectal cancer) CRC according to age, stage, and sex are presented in Fig. 2; Supplementary Table S3.

### Role of Biomarkers in Prognostic Effect of Primary Tumor Location

Since it has been hypothesized that certain biomarkers might play a role in the prognostic effects of primary tumor location, we studied additional data on biomarkers in stage IV CRC in the second part of the study. Biomarker data of 1035 patients with metastatic CRC (mCRC) diagnosed in 2014 and 1182 diagnosed in 2015 was collected. We excluded 348 patients with a second tumor and 51 patients with tumors located in the appendix or with overlapping or unspecified tumor locations. Supplementary Fig. 1 presents the study population selection process. The final study population consisted of 1818 patients with mCRC; 613 (33.7%) were located on the right side of the colon, 743 (40.9%) were located on the left side of the colon, and 462 (25.4%) rectal cancers. Table 3 provides an overview of the characteristics and biomarker status of the studied patients with mCRC. The median ­follow-up time in the study population was 17.0 months (until death, alive, or lost to follow-up). As expected, deficient mismatch repair status and *KRAS* and *BRAF* mutations were more frequent in right-sided CRC, while *NRAS* mutations were more frequent in left-sided CRC.

**Table 3.** Demographic characteristics and biomarker status of study population.

Variable^a^	All *n* = 832	Right-sided *n* = 268	Left-sided *n* = 352	Rectal *n* = 212
Median age in year (range)	68 (30-98)	70 (36-96)	68 (33-98)	65 (30-98)
Age >70 year	370 (44.5%)	137 (51.1%)	155 (44.0%)	78 (36.8%)
Age group				
0-59	202 (24.3%)	57 (21.3%)	79 (22.4%)	66 (31.1%)
60-69	260 (31.2%)	74 (27.6%)	118 (33.5%)	68 (32.1%)
70-79	238 (28.6%)	78 (29.1%)	103 (29.3%)	57 (26.9%)
>80	132 (15.9%)	59 (22.0%)	52 (14.8%)	21 (9.9%)
Male/female	487/345 Ratio 1.41	128/140 Ratio 0.91	214/138 Ratio 1.55	145/67 Ratio 2.16
KRAS				
*KRAS* WT	316 (51.5%)	86 (47.5%)	154 (57.2%)	76 (46.6%)
*KRAS* MT	297 (48.5%)	95 (52.5%)	115 (42.8%)	87 (53.4%)
KRAS not tested	219	87	83	49
NRAS				
*NRAS* WT	360 (93.7%)	99 (94.3%)	166 (93.3%)	95 (94.1%)
*NRAS* MT	24 (6.3%)	6 (5.7%)	12 (6.7%)	6 (5.9%)
NRAS not tested	448	163	174	111
BRAF				
*BRAF* WT	173 (86.5%)	41 (65.1%)	82 (94.3%)	50 (100%)
*BRAF* MT	27 (13.5%)	22 (34.9%)	5 (5.7%)	0 (0%)
BRAF not tested	632	205	265	162

Variable^b^	All *n* = 1818	Right-sided *n* = 613	Left-sided *n* = 743	Rectal *n* = 462
Median age in year (range)	68 (17-99)	71 (17-96)	68 (19-90)	65.5 (30-98)
Age >70 year	834	335	326	174
Age group				
0-59	462	126	196	140
60-69	521	152	221	148
70-79	499	179	205	115
>80	335	156	121	59
Male/female	1055/763 Ratio 1.38	304/309 Ratio 0.98	453/290 Ratio 1.56	298/164 Ratio 1.82
MMR				
dMMR	39 (6.2%)	28 (12.2%)	8 (3.1%)	3 (2.0%)
pMMR	595 (93.8%)	202 (87.8%)	246 (96.9%)	147 (98.0%)
MMR not tested	1184	383	489	312

^a^Diagnosed in 2014.

^b^Diagnosed in 2014 and 2015.

Abbreviations: DMMR, deficient mismatch repair status; MT, mutant type; PMMR, proficient mismatch repair status; WT, wild type.

When studying the effect of the covariates (sex and age) on survival separately in a Cox proportional hazards model, only age was significantly associated with survival and therefore included as a covariate in the following analyses (sex, hazard ratio (HR) = 0.98, *P* = .81; age, HR = 1.03, *P* = 1.78E-15).

**Figure 3.**
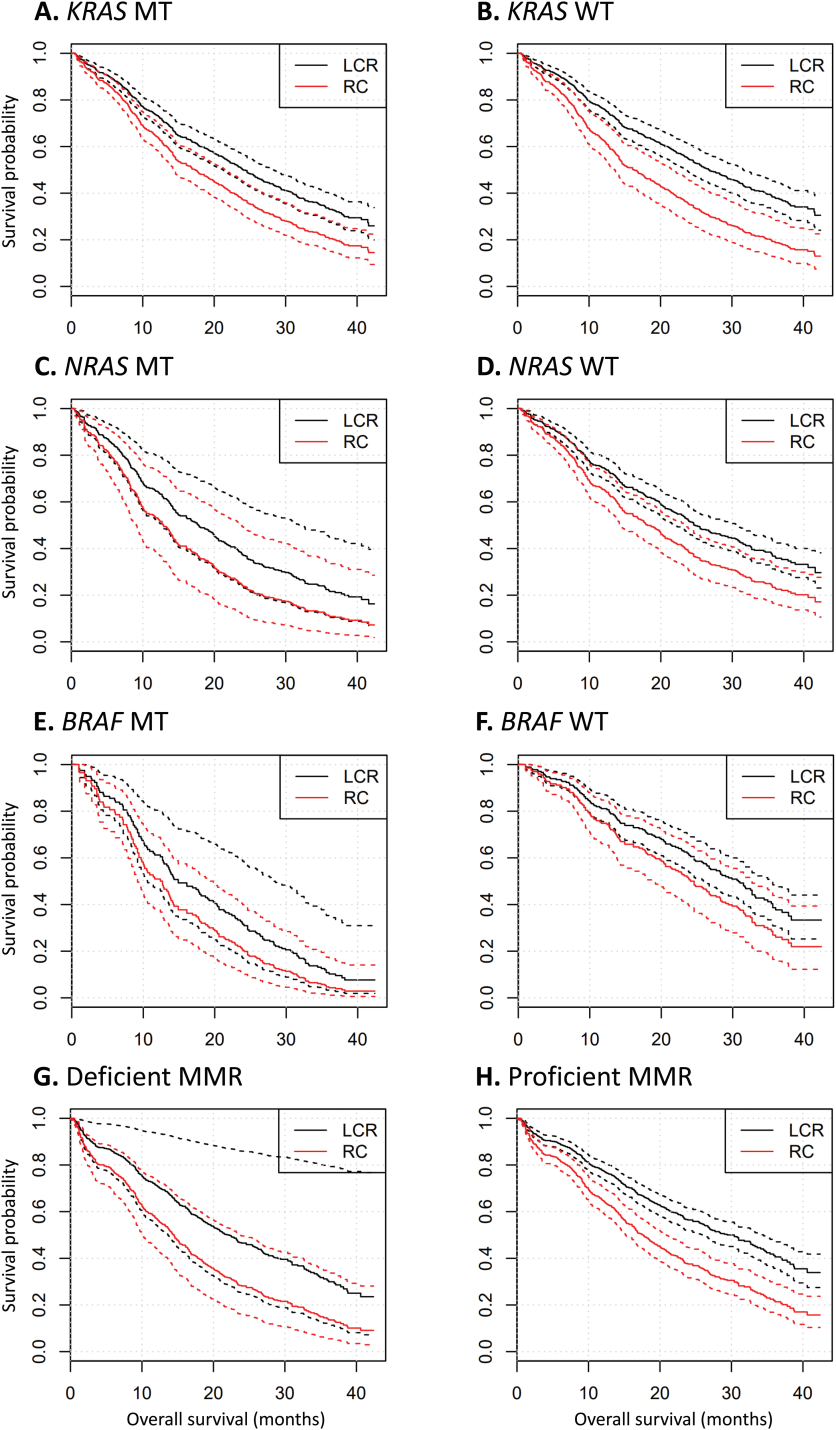
Kaplan-Meier curves of overall survival according to primary tumor location. Kaplan-Meier curves of overall survival according to mutational status, mismatch repair status and primary tumor location. Dotted lines represent 95% CIs. (**A**) All patients with *KRAS* mutant tumors, (**B**) all patients with *KRAS* wild-type tumors, (**C**) all patients with *NRAS* mutant tumors, (**D**) all patients with *NRAS* wild-type tumors, (**E**) all patients with *BRAF* mutant tumors, (**F**) all patients with *BRAF* wild-type tumors, (**G**) all patients with tumors with deficient mismatch repair status, (**H**) all patients with tumors with proficient mismatch repair status. Abbreviations: MT, mutant type; WT, wild-type; LCR, left-sided colon cancer and rectal cancer; RC, right-sided colon cancer.

First, we studied the main effect of the biomarkers on survival with primary tumor location and age as covariates in the model. *KRAS* and *NRAS* mutational status were not significantly associated with survival (respectively, *P* = .46 and *P* = .10). On the other hand, *BRAF* mutational status did show a significant association with survival (*P* = 8E-4) (Table 4). However, it should be noted that there was a very strong association between *BRAF* mutational status and location, with *BRAF* mutant-type tumors found more frequently in right-sided CRC (*P* = 7.1E−9). This strong association could lead to confounding when studying the effect of primary tumor location and the biomarker on survival. Likewise, MMR status had no significant association with survival (*P* = .20), when location and age were used as covariates in the model. However, it is important to note that there was a strong association between location and MMR status (*P* = 4.4E-6), with the deficient MMR status being more frequent among patients with right-sided cancer. Due to this multicollinearity, the effect of MMR status was not significant in the model that included location as covariate. Nonetheless, when only age was included as covariate, there was a marginally significant effect of MMR status (*P* = .03). Fig. 3; Supplementary Figs. S2–S10 show the Kaplan-Meier survival plots for each biomarker and location.

**Table 4.** Effects of location and biomarkers on survival.

			95% CI	
Variable	*P*-value	Hazard ratio	Lower limit	Upper limit
MMR status	0.20	1.30	0.87	1.96
*KRAS* status	0.46	1.08	0.88	1.30
*NRAS* status	0.10	1.49	0.92	2.44
*BRAF* status	0.0008	2.33	1.42	3.85
Location	0.11	1.38	0.93	2.04

Cox proportional hazard analysis adjusted for age and location (coded as 2-level variable). Proficient mismatch repair status, wild type, and left-sided combined with rectal location are coded as reference level.

Abbreviation: MMR, mismatch repair status.

Second, to study the impact of the biomarkers on the association between location and survival, we fitted Cox proportional hazard models with the main effects of each biomarker separately (either *KRAS, NRAS, or BRAF* mutational status or mismatch repair status) and location, and the interaction between them. The significance of the interaction term then indicates if the effect of the studied biomarker is uniform across all locations. The significance of these interaction terms was tested using a likelihood ratio test. In this analysis, no biomarker showed a significant interaction with location. Therefore, the location of the tumor did not influence the effect of the biomarkers on survival and the biomarker did not influence the effect of primary tumor location on survival (Supplementary Table S4).

Furthermore, primary tumor location remained highly significantly associated with survival in each model, independent of the covariates. Only in the model with *BRAF* mutational status, primary tumor location was not as strongly associated with survival (but still statistically significant), presumably because of the aforementioned partial confounding with *BRAF*.

All the aforementioned analyses were performed with primary tumor location coded as a 2-level variable (right versus left combined with rectal). When PTL was analyzed as a 3-level variable (right versus left vs rectal), similar results were obtained.

## Discussion

In the first analysis of this study, we observed a significantly better survival in left-sided CRC compared to right-sided CRC (when all stages, ages, and sexes were combined) in the Belgian population. This corresponds to the results of other population-based studies comparing survival in left- and right-sided CRC (Supplementary Table S5). In most of these population-based studies, right-sided CRC was associated with significantly poorer survival with hazard ratios ranging from 1.01 to 1.3 (in all stages combined).^4,5,17-27^ However, some studies reported no significant difference or even better survival in patients with right-sided CRC.^28-30^ This difference in results might be due to different definitions of right- and left-sided CRC, different study populations, or different statistical analyses.

A significant finding of the current study was that after stratification by stage, age, and sex, we noticed a better prognosis for left-sided CRC in almost every subgroup. Nonetheless, we observed the opposite association in 2 subgroups of patients: (i) patients of 80 years and older and (ii) patients with stage II disease.

In patients of 80 years and older a better survival was observed in right-sided CRC compared to left-sided CRC. This phenomenon was also observed in other studies; however, the mechanism behind this observation remains unclear.^17^

Our observation in stage II confirms the findings of other population-based studies that also observed better survival in right-sided compared to left-sided CRC.^5,17,21,27-31^ It has been suggested that better survival in right-sided stage II CRC is caused by a higher frequency of tumors with dMMR status or microsatellite instability (MSI) in this group. Tumors with MSI are associated with less metastasis and an overall better prognosis.^5^ However, the prognostic impact of MSI depends on stage. In stage II, tumors with MSI have a superior prognosis compared with microsatellite stable tumors. While in stage III the prognostic effect of MSI depends on the risk group. In the low-risk group (T1-3 and N1), MSI patients had a longer OS compared to the MSS group, whereas in the high-risk group (T4 or N2) similar survival was observed in the MSI and MSS group.^32,33^ In stage IV, a negative prognostic effect has been reported in univariate analysis, but it did not remain significant after multivariable adjustment. As a result, the prognostic effect of MSI in metastatic settings remains unclear.^33,34^

As indicated by the complicated relation among stage, MMR status, and prognosis, the relationship between PTL and prognosis in CRC is very complex and the identification of the factors that are responsible for the prognostic value of PTL is hampered by the many factors involved and the potential interaction between them. Therefore, in a second analysis in a group of stage IV CRC, we studied the role of biomarkers in the prognostic effect of PTL.

Consistent with other studies, right-sided CRC in our population harbored more frequently deficient MMR status and K*RAS* and *BRAF* mutations compared to left-sided tumors, while *NRAS* mutations were more frequent in left-sided CRC.^25,35-40^ Even though *BRAF* mutations are known to be associated with poor outcomes and are more frequent in right-sided CRC, our results indicate that right-sided CRC is still associated with significantly poorer survival regardless of *BRAF* mutational status. However, since there was a strong association between *BRAF* mutational status and PTL, we hypothesize that the higher frequency of *BRAF* mutations is at least partly responsible for the prognostic effect of PTL, but additional factors must be involved.

The other molecular markers, including MMR status and *KRAS* and *NRAS* mutational status, were not significantly associated with survival in the current study population. This might be due to the fact that only a small number of patients with tumors with dMMR status (*n* = 39) were included and therefore, the study was underpowered to detect a prognostic effect or interaction. Also, only a small number of *BRAF* MT (*n* = 27) patients was included, but even though the study may have been underpowered for this analysis a significant association could be detected. Previous studies also concluded that right-sided CRC was associated with poor survival, independent of the known prognostic factors (including *KRAS*, *BRAF*, and MMR status).^38,39^

The main strength of this study is the fact that it is based on high-quality information from a large population-based database. We included almost 92,000 patients in total, providing high power to the statistical analyses. Furthermore, we included all Belgian patients to create a heterogeneous group, covering all institutions and regions. Since this was an unselected population, this study was thus based on unbiased data. This is in contrast to other large cohort studies, which are mostly based on data from clinical trials. Second, we provided a long-term follow-up (January 2004-July 2017).

However, one of the limitations is the retrospective and observational character of the study. We considered numerous variables, which could at least in part explain the differences in survival between left- and right-sided CRC. However, other prognostic factors could not be examined (eg, comorbidity, disease bulk, location of metastasis, etc.) since those variables are not registered in the BCR. Second, we did not collect information on therapy. This would have been of particular interest since it has been proven that primary tumor location also has a predictive value.^2,41^ Post hoc analyses of clinical trials demonstrated an association between PTL and responsiveness to anti-EGFR therapy with patients with left-sided *KRAS* wild-type mCRC experiencing a significantly improved survival compared to patients with right-sided tumors.^10,13,31,42,43^ Another potential drawback of this study is the fact that only patients with synchronous metastatic disease were included. Since the BCR only receives data about stage at diagnosis and does not receive information concerning recurrence or metastasis during follow-up, only patients with the synchronous disease could be included. This might have caused a selection bias since the location and molecular characteristics can differ between metachronous and synchronous metastatic disease.^44^ Lastly, also the retrospective nature of our study might have led to a slight selection bias with respect to the biomarkers studied. We studied the biomarker status of 1818 patients, but not all biomarkers were tested in all patients (*KRAS* 613/832 (73.7%), *NRAS* 384/832 (46.2%), *BRAF* 200/832 (24.0%), and MMR status 634/1,818 (34.9%)). Since biomarker testing was mostly relevant for patients considered for anti-EGFR therapy (*KRAS, NRAS*) or younger patients (MMR testing for Lynch syndrome or *BRAF* testing for prognostication), it is possible that more young and fit patients with mCRC were tested. Furthermore, in 2014 and 2015, biomarker testing was not yet part of the standard work-up of every patient in every hospital. Therefore, it might be possible that biomarker status was tested more frequently in hospitals with specialized oncology clinics compared to smaller hospitals. Furthermore, the lack of standard techniques to test the biomarkers hampers interpretation.

Based on the strengths and limitations of our study, we can conclude that we adjusted for the most important patient- and cancer-related prognostic factors and that this study provides valuable data. To our knowledge, this is one of the largest population-based studies that include biomarker information.

## Conclusion

In summary, this Belgian population-based study on CRC confirms in an unselected population that relative survival is significantly higher in patients with left-sided CRC compared to right-sided CRC, in all stages and ages combined. However, in stage II and certain subgroups of patients over 80 years of age (stage I >80-year-old males, stage III >80-year–old males and females, and stage IV >80-year-old males) right-sided CRC shows a significantly better prognosis. Therefore, we can conclude that the prognostic effect of tumor location depends on age, sex, and stage in Belgian patients with CRC.

When studying the role of biomarkers in the prognostic value of PTL in 1818 Belgian patients with stage IV CRC, we found that none of the biomarkers (*NRAS, KRAS,* and *BRAF* mutational status and MMR) showed a significant interaction with location. Indicating that the effect of primary tumor location on survival was not influenced by these biomarkers in this population. Furthermore, it appears that in mCRC left-sided tumors are associated with a better survival compared to right-sided tumors, regardless of *RAS* and *BRAF* mutational status or mismatch repair status. However, the number of patients with deficient MMR or *BRAF* mutations in this population was too small to draw definitive conclusions.

Based on this study, the precise mechanism causing the difference in survival between left- and right-sided CRC remains to be elucidated. Further research should focus on determining these underlying complex molecular mechanisms.

## Supplementary Material

oyad074_suppl_Supplementary_Materials

## Data Availability

The data underlying this article will be shared on reasonable request to the corresponding author.

## References

[1] Bray F , Ferlay J, Soerjomataram I, et al. Global cancer statistics 2018: GLOBOCAN estimates of incidence and mortality worldwide for 36 cancers in 185 countries. CA Cancer J Clin. 2018;68(6):394-424. 10.3322/caac.21492. 30207593

[2] Boeckx N , Janssens K, Van Camp G, et al. The predictive value of primary tumor location in patients with metastatic colorectal cancer: a systematic review. Crit Rev Oncol Hematol. 2018;121(January):1-10. 10.1016/j.critrevonc.2017.11.003. 29279095

[3] Shen H , Yang J, Huang Q, et al. Different treatment strategies and molecular features between right-sided and left-sided colon cancers. World J Gastroenterol. 2015;21(21):6470-6478. 10.3748/wjg.v21.i21.6470. 26074686 PMC4458758

[4] Ahmed S , Pahwa P, Le D, et al. Primary tumor location and survival in the general population with metastatic colorectal cancer. Clin Colorectal Cancer. 2018;17(2):e201-e206. 10.1016/j.clcc.2017.11.001. 29221688

[5] Meguid RA , Slidell MB, Wolfgang CL, Chang DC, Ahuja N. Is there a difference in survival between right- versus left-sided colon cancers? Ann Surg Oncol. 2008;15(9):2388-2394. 10.1245/s10434-008-0015-y. 18622647 PMC3072702

[6] Bufill JA. Colorectal cancer: evidence for distinct genetic categories based on proximal or distal tumor location. Ann Intern Med. 1990;113(10):779-788. 10.7326/0003-4819-113-10-779. 2240880

[7] Holch JW , Ricard I, Stintzing S, Modest DP, Heinemann V. The relevance of primary tumour location in patients with metastatic ­colorectal cancer: a meta-analysis of first-line clinical trials. Eur J Cancer. 2017;70(January):87-98. 10.1016/j.ejca.2016.10.007. 27907852

[8] Sunakawa Y , Ichikawa W, Tsuji A, et al. Prognostic impact of primary tumor location on clinical outcomes of metastatic colorectal cancer treated with cetuximab plus oxaliplatin-based chemotherapy: a subgroup analysis of the JACCRO CC-05/06 Trials. Clin Colorectal Cancer. 2016;16.(3).):171-180. 10.1016/j.clcc.2016.09.010 27856123

[9] Lu HJ , Lin JK, Chen WS, et al. Primary tumor location is an important predictive factor for wild-type KRAS metastatic colon cancer treated with cetuximab as front-line bio-therapy. Asia Pac J Clin Oncol. 2016;12(3):207-215. 10.1111/ajco.12469. 26935130

[10] Tejpar S , Stintzing S, Ciardiello F, et al. Prognostic and predictive relevance of primary tumor location in patients with RAS ­wild-type metastatic colorectal cancer: retrospective analyses of the CRYSTAL and FIRE-3 trials. JAMA Oncol. 2017;3(2):194-201. 10.1001/jamaoncol.2016.3797 27722750 PMC7505121

[11] Loupakis F , Yang D, Yau L, et al. Primary tumor location as a prognostic factor in metastatic colorectal cancer. J Natl Cancer Inst. 2015;107(3):1-9. https://academic.oup.com/jnci/issue/107/3 10.1093/jnci/dju427 PMC4565528 25713148

[12] Miyamoto Y , Hayashi N, Sakamoto Y, et al. Predictors of long-term survival in patients with stage IV colorectal cancer with ­multi-organ metastases: a single-center retrospective analysis. Int J Clin Oncol. 2015;20(6):1140-1146. 10.1007/s10147-015-0835-2. 25947545

[13] Wang F , Bai L, Liu TS, et al. Right-sided colon cancer and left-sided colorectal cancers respond differently to cetuximab. Chin J Cancer. 2015;34(9):384-393. 10.1186/s40880-015-0022-x. 26111811 PMC4593341

[14] Petrelli F , Tomasello G, Borgonovo K, et al. Prognostic survival associated with left-sided vs right-sided colon cancer: a systematic review and meta-analysis. JAMA Oncol. 2017;3(2):211-219. 10.1001/jamaoncol.2016.4227 27787550

[15] Yahagi M , Okabayashi K, Hasegawa H, Tsuruta M, Kitagawa Y. The worse prognosis of right-sided compared with left-sided colon cancers: a systematic review and meta-analysis. J Gastrointest Surg. 2016;20(3):648-655. 10.1007/s11605-015-3026-6. 26573851

[16] Li P , Xiao Z, Braciak TA, et al. A relationship to survival is seen by combining the factors of mismatch repair status, tumor location and age of onset in colorectal cancer patients. PLoS One. 2017;12(3):e0172799. 10.1371/journal.pone.0172799. 28253296 PMC5333840

[17] Yang J , Du XL, Li ST, et al. Characteristics of differently located colorectal cancers support proximal and distal classification: a population-based study of 57,847 patients. PLoS One. 2016;11(12):e0167540. 27936129 10.1371/journal.pone.0167540 PMC5147913

[18] Gervaz P , Usel M, Rapiti E, et al. Right colon cancer: left behind. Eur J Surg Oncol. 2016;42(9):1343-1349. 10.1016/j.ejso.2016.04.002. 27178778

[19] Sharkas GF , Arqoub KH, Khader YS, et al. Colorectal cancer in Jordan: survival rate and its related factors. J Oncol. 2017;2017:3180762. 10.1155/2017/3180762. 28458690 PMC5387838

[20] Zheng C , Jiang F, Lin H, Li S. Clinical characteristics and prognosis of different primary tumor location in colorectal cancer: a ­population-based cohort study. Clin Transl Oncol. 2019;21(11):1524-1531. 10.1007/s12094-019-02083-1 30875062

[21] Nakagawa-Senda H , Hori M, Matsuda T, Ito H. Prognostic impact of tumor location in colon cancer: the monitoring of cancer incidence in Japan (MCIJ) project. BMC Cancer. 2019;19(1):431. 10.1186/s12885-019-5644-y. 31072372 PMC6509813

[22] Jess P , Hansen IO, Gamborg M, Jess T; Danish Colorectal Cancer G. A nationwide Danish cohort study challenging the categorisation into right-sided and left-sided colon cancer. BMJ Open. 2013;3(5):e002608. 10.1136/bmjopen-2013-002608. PMC3657647 23793665

[23] Brenner H , Bouvier AM, Foschi R, et al; EUROCARE Working Group. Progress in colorectal cancer survival in Europe from the late 1980s to the early 21st century: the EUROCARE study. Int J Cancer. 2012;131(7):1649-1658. 10.1002/ijc.26192. 21607946

[24] Zheng C , Jiang F, Lin H, Li S. Clinical characteristics and prognosis of different primary tumor location in colorectal cancer: a ­population-based cohort study. Clin Transl Oncol. 2019;21(11):1524-1531. 10.1007/s12094-019-02083-1. 30875062

[25] Narayanan S , Gabriel E, Attwood K, Boland P, Nurkin S. Association of clinicopathologic and molecular markers on stage-specific survival of right versus left colon cancer. Clin Colorectal Cancer. 2018;17(4):e671-e678. 10.1016/j.clcc.2018.07.001. 30108021 PMC10625797

[26] Wong R. Proximal tumors are associated with greater mortality in colon cancer. J Gen Intern Med. 2010;25(11):1157-1163. 10.1007/s11606-010-1460-4. 20652758 PMC2947632

[27] Ulanja MB , Rishi M, Beutler BD, et al. Colon cancer sidedness, presentation, and survival at different stages. J Oncol. 2019;2019:4315032. 10.1155/2019/4315032. 30915121 PMC6409047

[28] Brungs D , Aghmesheh M, de Souza P, et al. Sidedness is prognostic in locoregional colon cancer: an analysis of 9509 Australian patients. BMC Cancer. 2017;17(1):251. 10.1186/s12885-017-3255-z. 28390415 PMC5385038

[29] Weiss JM , Pfau PR, O’Connor ES, et al. Mortality by stage for right- versus left-sided colon cancer: analysis of surveillance, epidemiology, and end results—Medicare data. J Clin Oncol. 2011;29(33):4401-4409. 10.1200/JCO.2011.36.4414. 21969498 PMC3221523

[30] Warschkow R , Sulz MC, Marti L, et al. Better survival in right-sided versus left-sided stage I–III colon cancer patients. BMC Cancer. 2016;16(1):554. 10.1186/s12885-016-2412-0. 27464835 PMC4964057

[31] Li Y , Feng Y, Dai W, et al. Prognostic effect of tumor sidedness in colorectal cancer: A seer-based analysis. Clin Colorectal Cancer. 2019;18:e104-e116. 30448100 10.1016/j.clcc.2018.10.005

[32] Cohen R , Taieb J, Fiskum J, et al. Microsatellite instability in patients with stage III colon cancer receiving fluoropyrimidine with or without oxaliplatin: an ACCENT pooled analysis of 12 adjuvant trials. J Clin Oncol. 2021;39(6):642-651. 10.1200/JCO.20.01600. 33356421 PMC8189604

[33] Innocenti F , Ou FS, Qu X, et al. Mutational analysis of patients with colorectal cancer in CALGB/SWOG 80405 identifies new roles of microsatellite instability and tumor mutational burden for patient outcome. J Clin Oncol. 2019;37(14):1217-1227. 10.1200/JCO.18.01798. 30865548 PMC6506418

[34] Lee MS , Menter DG, Kopetz S. Right versus left colon cancer biology: integrating the consensus molecular subtypes. J Natl Compr Canc Netw. 2017;15(3):411-419. 10.6004/jnccn.2017.0038. 28275039

[35] Hsu YL , Lin CC, Jiang JK, et al. Clinicopathological and molecular differences in colorectal cancer according to location. Int J Biol Markers. 2019;34(1):47-53. 10.1177/1724600818807164. 30854932

[36] Cremolini C , Antoniotti C, Lonardi S, et al. Primary tumor sidedness and benefit from FOLFOXIRI plus bevacizumab as initial therapy for metastatic colorectal cancer. Ann Oncol. 2018;29(7):1528-1534. 10.1093/annonc/mdy140 29873679

[37] Shimada Y , Kameyama H, Nagahashi M, et al. Comprehensive genomic sequencing detects important genetic differences between right-sided and left-sided colorectal cancer. Oncotarget. 2017;8(55):93567-93579. 10.18632/oncotarget.20510. 29212173 PMC5706819

[38] Mendis S , Beck S, Lee B, et al. Right versus left sided metastatic colorectal cancer: teasing out clinicopathologic drivers of disparity in survival. Asia Pac J Clin Oncol. 2019;15(3):136-143. 10.1111/ajco.13135. 30761750

[39] Xie MZ , Li JL, Cai ZM, Li KZ, Hu BL. Impact of primary colorectal location on the KRAS status and its prognostic value. BMC Gastroenterol. 2019;19(1):46. 10.1186/s12876-019-0965-5. 30917791 PMC6437985

[40] Price TJ , Beeke C, Townsend AR, et al. BRAF mutation testing and metastatic colorectal cancer in the community setting: is there an urgent need for more education? Mol Diagn Ther. 2016;20(1):75-82. 10.1007/s40291-015-0179-7. 26714964

[41] Personeni N , Smiroldo V, Giunta EF, et al. Tackling refractory metastatic colorectal cancer: future perspectives. Cancers (Basel). 2021;13(18):1-20. 10.3390/cancers13184506 PMC8472765 34572729

[42] Boeckx N , Koukakis R, Op de Beeck K, et al. Effect of primary tumor location on second- or later-line treatment outcomes in patients with RAS wild-type metastatic colorectal cancer and all treatment lines in patients with RAS mutations in four randomized panitumumab studies. Clin Colorectal Cancer. 2018;17(3):170-178.e3. 10.1016/j.clcc.2018.03.005. 29627309

[43] Kennecke HF , Yin Y, Davies JM, et al. Prognostic effect of sidedness in early stage versus advanced colon cancer. Health Sci Rep. 2018;1(8):e54. 10.1002/hsr2.54. 30623090 PMC6266477

[44] Lan YT , Chang SC, Lin PC, et al. Clinicopathological and molecular features between synchronous and metachronous metastases in colorectal cancer. Am J Cancer Res. 2021;11(4):1646-1658. 33948379 PMC8085873

