# Supplementary material for: A Belgian Population-Based Study Reveals Subgroups of Right-sided Colorectal Cancer with a Better Prognosis Compared to Left-sided Cancer
Source: Oncologist. 2023 Apr 18;28(6):e331–40. doi: 10.1093/oncolo/oyad074 (PMC10243787; doi:10.1093/oncolo/oyad074)
Supplement: oyad074_suppl_Supplementary_Materials [file oyad074_suppl_supplementary_materials.zip › oyad074_suppl_Supplementary_Figure_Tables.docx]

## Supplemental Table S1. Odds ratios comparing right- to left-sided (incl. rectal cancer) CRC according to stage.

| Stage | Odds ratio | 95% Confidence interval |
| --- | --- | --- |
| I | 1.49 | 1.31-1.71 |
| II | 0.51 | 0.47-0.55 |
| III | 1.21 | 1.14-1.28 |
| IV | 1.58 | 1.44-1.74 |

p < 0.05 for all analyses.

The odds ratio was defined as the odds to die of cancer during 5-year follow-up in the non-reference category (right-sided CRC) versus the reference category (left-sided CRC).

## Supplemental Table S2. Odds ratios comparing right- to left-sided CRC (excl. rectal cancer) according to stage

| Stage | Odds ratio | 95% Confidence interval |
| --- | --- | --- |
| I | 1.60 | 1.38-1.87 |
| II | 0.64 | 0.58-0.69 |
| III | 1.37 | 1.28-1.46 |
| IV | 1.57 | 1.41-1.74 |

p < 0.05 for all analyses.

The odds ratio was defined as the odds to die of cancer during 5-year follow-up in the non-reference category (right-sided CRC) versus the reference category (left-sided CRC).

## Supplemental table S3. Odds ratios comparing right- to left-sided (incl. rectal cancer) CRC according to stage, age and sex

| **Location** | **Stage** | **Age** | **OR male** | **95% CI M** | **OR female** | **95% CI** |
| --- | --- | --- | --- | --- | --- | --- |
| LCR | I | 0-59 | 1 (reference) | 0.726-1.377 | 1 (reference) | 0.564-1.773 |
| RC | I | 0-59 | 1.921 | 1.162-3.178 | 1.631 | 0.656-4.054 |
| LCR | I | 60-69 | 1.299 | 0.988-1.709 | 1.648 | 1.009-2.692 |
| RC | I | 60-69 | 2.416 | 1.691-3.453 | 3.647 | 2.075-6.412 |
| LCR | I | 70-79 | 1.335 | 1.020-1.749 | 2.020 | 1.271-3.211 |
| RC | I | 70-79 | 1.621 | 1.148-2.287 | 4.778 | 2.959-7.716 |
| LCR | I | 80+ | 2.439 | 1.842-3.228 | 2.023 | 1.245-3.288 |
| RC | I | 80+ | 1.713 | 1.164-2.519 | 3.121 | 1.897-5.135 |
| LC | II | 0-59 | 2.222 | 1.679-2.941 | 5.095 | 3.256-7.974 |
| RC | II | 0-59 | 1.891 | 1.316-2.716 | 3.756 | 2.198-6.416 |
| LCR | II | 60-69 | 2.868 | 2.224-3.698 | 5.848 | 3.784-9.039 |
| RC | II | 60-69 | 2.646 | 1.970-3.556 | 5.282 | 3.346-8.337 |
| LCR | II | 70-79 | 4.105 | 3.218-5.235 | 8.731 | 5.736-13.290 |
| RC | II | 70-79 | 2.884 | 2.208-3.768 | 3.442 | 2.206-5.372 |
| LCR | II | 80+ | 5.670 | 4.424-7.268 | 14.727 | 9.710-22.338 |
| RC | II | 80+ | 1.102 | 0.796-1.525 | 4.170 | 2.714-6.408 |
| LCR | III | 0-59 | 6.004 | 4.684-7.697 | 11.108 | 7.274-16.964 |
| RC | III | 0-59 | 6.062 | 4.470-8.220 | 18.968 | 12.026-29.916 |
| LCR | III | 60-69 | 6.289 | 4.935-8.015 | 12.581 | 8.256-19.171 |
| RC | III | 60-69 | 8.549 | 6.538-11.178 | 14.688 | 9.496-22.719 |
| LCR | III | 70-79 | 9.784 | 7.708-12.419 | 19.801 | 13.069-30.001 |
| RC | III | 70-79 | 10.224 | 7.929-13.183 | 25.955 | 17.064-39.478 |
| LCR | III | 80+ | 18.085 | 14.156-23.106 | 42.517 | 28.052-64.443 |
| RC | III | 80+ | 16.814 | 12.969-21.800 | 30.535 | 20.143-46.287 |
| LCR | IV | 0-59 | 49.374 | 38.316-63.624 | 100.607 | 65.752-153.939 |
| RC | IV | 0-59 | 93.805 | 66.252-132.817 | 185.294 | 115.191-298.060 |
| LCR | IV | 60-69 | 64.206 | 50.016-82.422 | 126.465 | 82.464-193.942 |
| RC | IV | 60-69 | 100.149 | 73.633-136.214 | 194.102 | 122.120-308.514 |
| LCR | IV | 70-79 | 106.548 | 82.448-137.692 | 200.443 | 130.301-308.344 |
| RC | IV | 70-79 | 133.454 | 98.326-181.133 | 292.758 | 185.753-461.405 |
| LCR | IV | 80+ | 226.149 | 163.321-313.148 | 489.511 | 306.157-782.675 |
| RC | IV | 80+ | 146.982 | 104.125-207.479 | 656.965 | 394.951-1,092.802 |

Five-year relative survival in stage 1 left-sided colon and rectal cancer patients aged 0 to 59 year was used as reference survival.

Abbreviations: OR, Odds ratio; CI, Confidence Interval; LCR, left-sided colon and rectal cancer; RC, right-sided colon cancer.

## Supplemental Table S4. Likelihood ratio test to check significance of interaction term between biomarker and location.

1. Primary tumor location coded as two-level variable (Right/Left combined with rectal)

| Variable | p-value | *X*^2^ | DF |
| --- | --- | --- | --- |
| MMR | 0.95 | 0.0047 | 1 |
| *KRAS* | 0.09 | 2.9033 | 1 |
| *NRAS* | 0.91 | 0.0134 | 1 |
| *BRAF* | 0.96 | 0.002 | 1 |

1. Primary tumor location coded as three-level variable (Right/Left/Rectal)

| Variable | p-value | *X*^2^ | df |
| --- | --- | --- | --- |
| MMR | 0.59 | 1.0405 | 2 |
| *KRAS* | 0.26 | 2.6905 | 2 |
| *NRAS* | 0.69 | 0.7423 | 2 |
| *BRAF* | 0.90 | 0.0147 | 1 |

Abbreviations: DF, Degrees of freedom; MMR, mismatch repair status

## Supplemental Table S5. Population-based studies regarding the prognostic value of primary tumor location

| Population-based studies on the prognostic value of primary tumor location | | | | | | | | |  |
| --- | --- | --- | --- | --- | --- | --- | --- | --- | --- |
| Author and year | **Database** | **Country** | **Date of diagnosis** | **Number of patients** | **Inclusion criteria** | **Location definition** | **Conclusion** |  |  |
| Meguid et al., 2008[5] | SEER | USA | 1988-2002 | 77,978 | >18y, surgery with curative intent for pathologically confirmed adenocarcinoma, exclusion of pre- or postoperative radiotherapy | Right; cecum-transverse colon (excl. appendix). Left; splenic flexure to sigmoid (excl. rectum and rectosigmoid junction). | Adjusted Cox regression: |  |  |
|  |  |  |  |  |  |  | All stages: right: HR = 1.042; 95% CI 1.02–1.07); p = 0.001 | R<L |  |
|  |  |  |  |  |  |  | Stage I: right: HR = 1.003; 95% CI 0.93-1.08, P = .93 | R=L |  |
|  |  |  |  |  |  |  | Stage II: right: HR = .91; 95% CI 0.88-0.95, P<.001 | R>L |  |
|  |  |  |  |  |  |  | Stage III: right: HR 1.06; 95% CI 1.02-1.11 ^a^ | R<L |  |
|  |  |  |  |  |  |  | Stage IV: right: HR 1.22; 95% CI 1.15-1.28 ^a^ | R<L |  |
| Wong et al., 2010[26] | SEER | USA | 1973-2005 | NA | all CRC | Right; cecum, appendix, ascending colon, hepatic flexure, and transverse colon. Left; splenic flexure, descending colon, sigmoid colon, rectosigmoid junction. Excl. rectum. | Multivariate logistic regression (5-year survival): |  |  |
|  |  |  |  |  |  |  | All stages: 1983-1997: Right: OR 0.88; 95% CI, 0.86–0.90 ^a^ | R<L |  |
|  |  |  |  |  |  |  | All stages: 1998-2005: Right: OR 0.87; 95% CI, 0.82–0.91 ^a^ | R<L |  |
|  |  |  |  |  |  |  | Localized disease: 1983-1997: Right: OR 0.84; 95% CI, 0.81–0.86 ^a^ | R<L |  |
|  |  |  |  |  |  |  | Localized disease: 1998-2005: Right: OR 0.85; 95% CI, 0.78–0.92 ^a^ | R<L |  |
|  |  |  |  |  |  |  |  |  |  |
| Weiss et al., 2011[29] | SEER | USA | 1992-2005 | 53,801 | ≥66y, stage I-III adenocarcinoma, surgery with curative intent, not mucinous cystadenocarcinoma | Right; cecum, ascending colon, hepatic ﬂexure and transverse colon. Left; splenic ﬂexure, descending colon, sigmoid colon, rectosigmoid (excl. rectal). | Adjusted Cox regression: |  |  |
|  |  |  |  |  |  |  | All stages: right HR, 1.01; 95% CI, 0.98-1.04; P=.598 | R=L |  |
|  |  |  |  |  |  |  | Stage I: right HR 0.95; 95%CI, 0.88-1.03; P=.211 | R=L |  |
|  |  |  |  |  |  |  | Stage II: right HR, 0.92; 95%CI,0.87-0.97; P<.001 | R>L |  |
|  |  |  |  |  |  |  | Stage III: right HR, 1.12; 95% CI, 1.06-1.18; P<.001 | R<L |  |
| Brenner et al., 2012[23] | EURO- | Europe | 1984-2002 | 696,997^c^ | 15-99y | Right; cecum to transverse colon. Left colon; splenic flexure to sigmoid colon. (Excl. Rectosigmoid junction and rectum) | Multivariate analysis (relative excess mortality):^b^ |  |  |
|  | CARE-4 |  |  |  |  |  | All stages; Left: HR 0.90; 95% CI 0.85–0.95 | R<L |  |
| Van der Pool et al. 2012[43] | Rotterdam Cancer Registry | Netherlands | 1995-2007 | 3482 | stage IV CRC | Right; NA. Left; splenic flexure and sigmoid. (excl. rectum) | Adjusted Cox regression: |  |  |
|  |  |  |  |  |  |  | Stage IV: Right: HR 1.22 (95% CI 1.12-1.32) ^a^ | R<L |  |
|  |  |  |  |  |  |  | All stages: Right: HR men 1.31, 95% CI 1.24 to 1.40. ^a^ | R<L |  |
| Price et al., 2015[44] | South Australian mCRC registry | South Australia | 2006 | 2,972 | only mCRC | Right; cecum to transverse colon. Left; splenic flexure to rectum, incl. rectum. | Adjusted Cox regression: |  |  |
|  |  |  |  |  |  |  | Stage IV: Right HR 1.25; 95% CI, 1.14-1.37; P ≤.001 | R<L |  |
| Gervaz et al., 2016[18] | Geneva Cancer Registry | Switzerland | 1980-2006 | 3,396 | all invasive adenocarcinoma cancers of the colon | Right: cecum, ascending colon, hepatic flexure, and transverse colon. Left; splenic flexure, descending colon, sigmoid, and recto-sigmoid junction. | Adjusted Cox regression (CRC specific 5-year survival): |  |  |
|  |  |  |  |  |  |  | All stages: Right: HR 1.25, 95% CI: 1.12-1.39, p < 0.001 | R<L |  |
| Warschkow et al., 2016[30] | SEER | USA | 2004-2012 | 91,146 | stage I-III | Right; cecum and ascending colon. Left; descending or sigmoid colon. | Adjusted Cox regression (cancer-specific mortality): |  |  |
|  |  |  |  |  |  |  | All stages: Right: HR = 1.03; 95% CI 0.99–1.08; p = 0.0156 | R=L |  |
|  |  |  |  |  |  |  | Propensity score matched analysis (cancer-specific mortality): |  |  |
|  |  |  |  |  |  |  | All stages: Right: HR = 0.90; 95% CI 0.87–0.93; p < 0.001 | R>L |  |
|  |  |  |  |  |  |  | Stage I: Right HR=0.71, 95 % CI:0.64−0.79, p < 0.001 | R>L |  |
|  |  |  |  |  |  |  | Stage II: Right HR=0.75, 95 % CI:0.70–0.80, p< 0.001 | R>L |  |
|  |  |  |  |  |  |  | Stage III: Right HR=1.04, 95 % CI:0.99–1.09, p = 0.129 | R=L |  |
| Yang et al. 2016[17] | SEER | USA | 2000-2012 | 57,847 | pathologically confirmed colorectal adenocarcinoma | Right; cecum, ascending colon, hepatic flexure and transverse colon. Left; splenic flexure, descending colon, sigmoid colon and rectosigmoid junction. (Excl. rectum) | Adjusted Cox regression (Disease specific survival): ^b^ |  |  |
|  |  |  |  |  |  |  | All stages: Left: HR 0.87, 95%CI 0.84-0.90, p <0.0001 | R<L |  |
|  |  |  |  |  |  |  | Stage I: Left: HR 0.81, 95% CI 0.69-0.93, p = 0.004 | R<L |  |
|  |  |  |  |  |  |  | Stage II: Left: HR 1.29, 95% CI 1.18-1.40, p < 0.0001 | R>L |  |
|  |  |  |  |  |  |  | Stage III: Left: HR 0.81, 95% CI 0.76-0.87, p < 0.0001 | R<L |  |
|  |  |  |  |  |  |  | Stage IV: Left: HR 0.77, 95% CI 0.72-0.81, p < 0.0001 | R<L |  |
| Brungs et al., 2017[28] | New South Wales cancer registry | Australia | 2006-2013 | 9,509 | stage I – III primary adenocarcinoma, no rectal cancer | Right; caecum to transverse colon. Left; splenic flexure to rectosigmoid. | Adjusted Cox regression (Cancer specific 5-year survival): |  |  |
|  |  |  |  |  |  |  | Overall: right HR 0.84, 95% CI 0.73–0.96, p = 0.011 | R>L |  |
|  |  |  |  |  |  |  | Stage I: right HR 0.51 95% CI 0.35–0.75, p = 0.0006 | R>L |  |
|  |  |  |  |  |  |  | Stage II: right HR 0.59 95% CI 0.45–0.78, p = 0.0002 | R>L |  |
|  |  |  |  |  |  |  | Stage III: right HR 1.12 95% CI 0.94–1.33, p = 0.22 | R<L |  |
| Ahmed et al., 2017[4] | Saskatchewan Cancer Registry | Saskatchewan province, Canada | 1992-2010 | 1,947 | adenocarcinoma stage 4 CRC, not neuroendocrine tumors, melanoma, lymphoma, GIST, and other histologic malignant diagnoses of colon and rectum | Right; ascending and transverse colonic tumors (up to the splenic flexure). Left; descending colon, sigmoid colon, and rectum. | Adjusted Cox regression: |  |  |
|  |  |  |  |  |  |  | Stage IV: Right HR 1.40, 95% CI 1.20-1.60, p <.001 | R<L |  |
| Sharkas et al., 2017[19] | Jordan cancer registry | Jordan | 2005-2010 | 3,005 | All CRC | Right; cecum, ascending colon up to the hepatic flexure. Left; splenic flexure and distal to the splenic flexure, including the rectum. | Adjusted Cox regression: |  |  |
|  |  |  |  |  |  |  | All stages: right: HR=1.3, 95%CI 1.1-1.6, p = 0.013 | R<L |  |
| Li et al. 2018[31] | SEER | USA | 2000-2014 | 238,826 | Surgery-performed, pathologically confirmed CRC | Right; cecum, ascending colon, hepatic flexure and transverse colon. Left; splenic flexure, descending colon, sigmoid and rectosigmoid. (Excl. rectum) | Adjusted Cox regression (Cause specific survival): ^b^ |  |  |
|  |  |  |  |  |  |  | Stage I-II: Left: HR 1.091, CI 1.052-1.132, P <0.001 | R>L |  |
|  |  |  |  |  |  |  | Stage III: Left HR 0.799, 95%CI 0.774-0.825, P<0.001 | R<L |  |
|  |  |  |  |  |  |  | Stage IV: Left HR 0.755, 95% CI 0.734-0.777, P<0.001 | R<L |  |
| Narayanan et al. 2018[25] | National Cancer Database | USA | 2004-2013 | 422,443 | all histologic colon adenocarcinoma | Right; cecum, ascending colon and hepatic flexure. Left; splenic flexure, descending colon, and sigmoid. Excl. appendiceal, transverse colon, rectosigmoid, and rectal. | Propensity adjusted Cox regression: |  |  |
|  |  |  |  |  |  |  | All stages: Right: HR 1.06, CI 1.04-1.09, p<0.001. | R<L |  |
| Mendis et al. 2019[36] | Treatment of Recurrent and Advanced Colorectal Cancer (TRACC) Registry | Australia and Hong Kong | 2009-2018 | 2,306 | stage IV CRC | Right; proximal to splenic flexure. Left; distal to the splenic flexure and incl. rectal cancers. | Adjusted Cox regression: |  |  |
|  |  |  |  |  |  |  | Stage IV: Right HR 1.55, CI 1.25-1.92, P < 0.001. | R<L |  |
| Nakagawa-Senda et al., 2019[21] | Monitoring of Cancer Incidence in Japan (MCIJ) project | Japan | 2006-2008 | 62,350 | all stages | Right; cecum, appendix, ascending colon, hepatic flexure and transversal colon. Left; splenic flexure, descending colon and sigmoid. | Adjusted Cox regression: |  |  |
|  |  |  |  |  |  |  | All stages: Right: Excess HR 1.20 (95% CI, 1.16–1.25), p = <0.001 | R<L |  |
|  |  |  |  |  |  |  | Localized stage: Right: Excess HR 0.74 (95% CI, 0.60-0.90) ^a^ | R>L |  |
|  |  |  |  |  |  |  | Regional stage: Right: Excess HR 1.25 (95% CI, 1.17–1.34) ^a^ | R<L |  |
|  |  |  |  |  |  |  | Distant stage: Right: Excess HR 1.20 (95% CI, 1.15–1.25) ^a^ | R<L |  |
| ULANYA ET AL. 2019[27] | SEER | USA | 2007-2015 | 163,980 | CRC | Right; cecum, ascending colon, hepatic flexure, transverse colon. Left; splenic flexure, descending, sigmoid colon, and rectosigmoid junction. Excl. Rectum. | Adjusted Cox regression (Cancer specific Survival):  All Stages: Left aHR =0.87; 95%CI 0.85-0.89, p<0.001  Stage II: Left aHR=1.30, 95%CI1.23-1.38, p<0.001  Stage III: Left aHR=0.84; 95%CI 0.800.87, p<0.001  Stage IV: Left aHR=0.79;95%CI0.77-0.81,p<0.001 | R<L  R>L  R<L  R<L |  |
| Zheng et al. 2019[24] | SEER | USA | 2006-2015 | 248,861 | CRC | Right; cecum, ascending colon, hepatic flexure, transverse colon. Left; splenic flexure, descending colon, sigmoid, rectosigmoid junction, and rectum. | Adjusted Cox regression: |  |  |
|  |  |  |  |  |  |  | Stages 0-IV: Right HR 1.224, 95% CI, 1.208–1.241, P<0.001 | R<L |  |
|  |  |  |  |  |  |  | Stage 0: Right HR 1.306, 95% CI, 1.171–1.457, P<0.001 | R<L |  |
|  |  |  |  |  |  |  | Stage I: Right HR 1.269, 95% CI 1.220–1.319, P<0.001 | R<L |  |
|  |  |  |  |  |  |  | Stage II: Right HR 1.042, 95% CI, 1.009–1.075, P 0.011 | R<L |  |
|  |  |  |  |  |  |  | Stage III: Right HR 1.501, 95% CI, 1.461–1.542, P<0.001 | R<L |  |
|  |  |  |  |  |  |  | Stage IV: Right HR 1.356, 95% CI, 1.328–1.385, P<0.001 | R<L |  |
| Population-based studies on the prognostic value of primary tumor location and biomarkers | | | | | | | | |  |
| Author and year | **Database** | **Country** | **Date of diagnosis** | **Number of patients** | **Inclusion criteria** | **Location definition** | **Conclusion** |  |  |
| Charlton et al., 2017[45] | SEER | USA | 2010-2012 | 16,952 | Microscopically confirmed stage IV adenocarcinoma of the colon. | Right; cecum, ascending colon, hepatic flexure, transverse. Left; splenic flexure, descending colon, sigmoid, rectosigmoid junction, excl. rectum. | Adjusted Cox-regression analyses: |  | |
|  |  |  |  |  |  |  | Stage IV Right: HR 1.27; 95% CI, 1.22–1.32^a^ | R<L | |
|  |  |  |  |  |  |  | Stage IV: left and right: *KRAS* mutational status no association with risk of death. |  |  |
|  |  |  |  |  |  |  | Stage IV: Right: *KRAS* MT no greater risk of death HR, 0.93; 95% CI, 0.83–1.03. |  |  |
|  |  |  |  |  |  |  | Stage IV: Left: *KRAS* MT greater risk of death HR, 1.18; 95% CI, 1.05–1.33. ^a^ |  |  |
|  |  |  |  |  |  |  | Stage IV: left and right: *KRAS* mutational status not prognostic. |  |  |

^a^Significant, if p-value not mentioned.

^b^ Reference is always left-sided CRC, except for ^b^.

^c^ = but only patients diagnosed in 2000-2002 from registries with >75% stage information used for this analysis = only 6 out of 25 registries

Abbreviations: CI, Confidence Interval
