# Supplementary material for: A Belgian Population-Based Study Reveals Subgroups of Right-sided Colorectal Cancer with a Better Prognosis Compared to Left-sided Cancer
Source: Oncologist. 2023 Apr 18;28(6):e331–40. doi: 10.1093/oncolo/oyad074 (PMC10243787; doi:10.1093/oncolo/oyad074)
Supplement: oyad074_suppl_Supplementary_Materials [file oyad074_suppl_supplementary_materials.zip › oyad074_suppl_Supplementary_Figure_Captions.docx]

**Supplemental Figure Captions**

**Supplemental Figure 1.** Patient selection flow chart.

Abbreviations: mCRC, metastatic colorectal cancer; MMR, mismatch repair status.

**Supplemental Figure 2.** Kaplan Meier curves of overall survival according to KRAS mutational status and primary tumor location. Dotted lines represent 95% confidence intervals. (A) In all patients with KRAS mutant type tumors, (B) all patients with KRAS wild-type tumors, (C) all patients with left-sided and rectal cancer, (D) all patients with right-sided colorectal cancer.

Abbreviations: MT, mutant type; WT, wild-type; LCR, left-sided colon cancer and rectal cancer; RC, right-sided colon cancer; CRC, colorectal cancer.

**Supplemental Figure 3.** Kaplan Meier curves of overall survival according to NRAS mutational status and primary tumor location. Dotted lines represent 95% confidence intervals. (A) In all patients with NRAS mutant type tumors, (B) all patients with NRAS wild-type tumors, (C) all patients with left-sided and rectal cancer, (D) all patients with right-sided colorectal cancer.

Abbreviations: MT, mutant type; WT, wild-type; LCR, left-sided colon cancer and rectal cancer; RC, right-sided colon cancer; CRC, colorectal cancer.

**Supplemental Figure 4.** Kaplan Meier curves of overall survival according to BRAF mutational status and primary tumor location. Dotted lines represent 95% confidence intervals. (A) In all patients with BRAF mutant type tumors, (B) all patients with BRAF wild-type tumors, (C) all patients with left-sided and rectal cancer, (D) all patients with right-sided colorectal cancer. Because of the small number of left-sided CRC patients with BRAF mutant type, the confidence interval in this subgroup is very large. Abbreviations: MT, mutant type; WT, wild-type; LCR, left-sided colon cancer and rectal cancer; RC, right-sided colon cancer; CRC, colorectal cancer.

**Supplemental Figure 5.** Kaplan Meier curves of overall survival according to mismatch repair status and primary tumor location. Dotted lines represent 95% confidence intervals. (A) In all patients with tumors with deficient mismatch repair status, (B) all patients with tumors with proficient mismatch repair status, (C) all patients with left-sided and rectal cancer, (D) all patients with right-sided colorectal cancer. Because of the small number of left-sided CRC patients with deficient mismatch repair status, the confidence interval in this subgroup is very large.

Abbreviations: MMR, mismatch repair; dMMR, deficient MMR; pMMR, proficient MMR; LCR, left-sided colon cancer and rectal cancer; RC, right-sided colon cancer; CRC, colorectal cancer.

**Supplemental Figure 6.** Kaplan Meier of overall survival according to primary tumor location in the mismatch proficient RAS mutant population. Dotted lines represent 95% confidence intervals.

In the proficient MMR and RAS mutant type population (n=102, 92 KRAS MT and 10 NRAS MT), primary tumor location (two-level) is not statistically significantly associated with overall survival (p=0.77, in a Cox proportional hazard analysis with age as covariate). Median overall survival is 26.9 months in the left-sided and rectal group and 21.2 months in the right-sided group.

**Supplemental Figure 7.** Kaplan Meier of overall survival according to primary tumor location in the mismatch proficient BRAF mutant population. Dotted lines represent 95% confidence intervals.

In the proficient MMR status and BRAF mutant type population (n=15, 4 left-sided and 11 right-sided), primary tumor location (two-level) is not statistically significantly associated with overall survival (p=0.92, in a Cox proportional hazard analysis with age as covariate). Median overall survival is 8.6 months in the left-sided and rectal group and 16.9 months in the right-sided group. Because of the small BRAF MT population, no conclusions can be drawn from these results.

**Supplemental Figure 8.** Kaplan Meier of overall survival according to primary tumor location in the mismatch proficient RAS and BRAF wild-type population. Dotted lines represent 95% confidence intervals.

In the mismatch repair proficient and BRAF and RAS wild-type population (n=54, 43 left-sided and 11 right-sided), primary tumor location (two-level) is not statistically significant associated with overall survival (p=0.50, in a Cox proportional hazard analysis with age as covariate). Median overall survival is 32.7 months in the left-sided and rectal group and 29.4 months in the right-sided group.

**Supplemental Figure 9.** Kaplan Meier of overall survival according to primary tumor location in the mismatch deficient population. Dotted lines represent 95% confidence intervals.

In the mismatch repair deficient population (n=39, with 2 KRAS MT and 4 BRAF MT, 11 left-sided and 28 right-sided), primary tumor location (two-level) is not statistically significant associated with overall survival (p=0.31, in a Cox proportional hazard analysis with age as covariate). Median overall survival is 16.8 months in the left-sided and rectal group and 9.0 months in the right-sided group.

**Supplemental Figure 10.** Kaplan Meier of overall survival according to primary tumor location in the mismatch deficient and RAS and BRAF wild-type population. Dotted lines represent 95% confidence intervals.

In the mismatch repair deficient and RAS and BRAF wild-type population (n=33, 9 left-sided and 24 right-sided), primary tumor location (two-level) is not statistically significantly associated with overall survival (p=0.45, in a Cox proportional hazard analysis with age as covariate). Median overall survival is 16.8 months in the left-sided and rectal group and 8.7 months in the right-sided group.
