# Supplementary figures and images for: A Belgian Population-Based Study Reveals Subgroups of Right-sided Colorectal Cancer with a Better Prognosis Compared to Left-sided Cancer
Source: Oncologist. 2023 Apr 18;28(6):e331–40. doi: 10.1093/oncolo/oyad074 (PMC10243787; doi:10.1093/oncolo/oyad074)

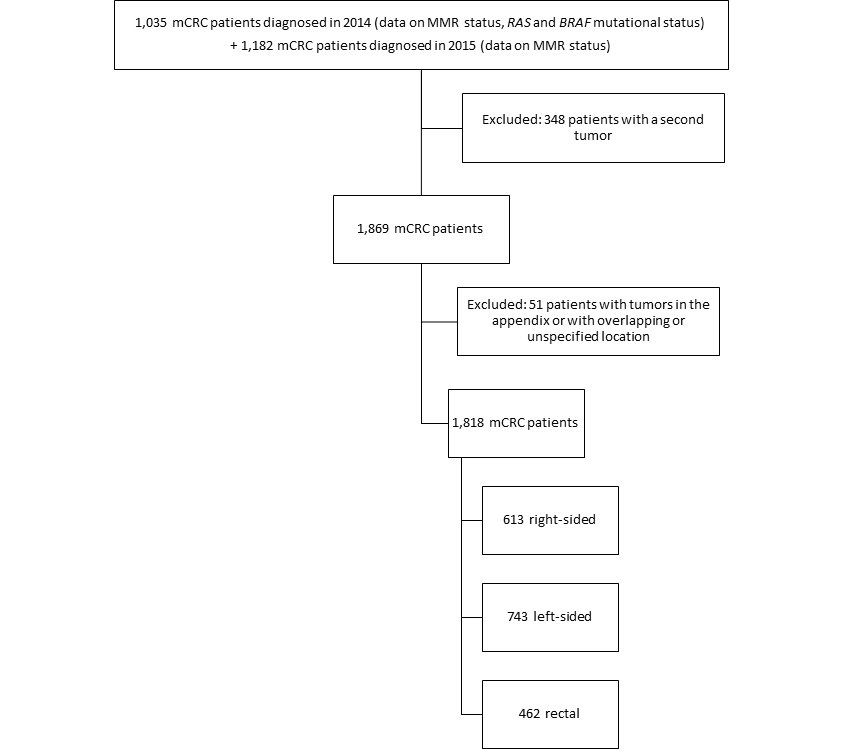

Supplement: oyad074_suppl_Supplementary_Materials [file oyad074_suppl_supplementary_materials.zip › oyad074_suppl_Supplementary_Figure_S1.tif]

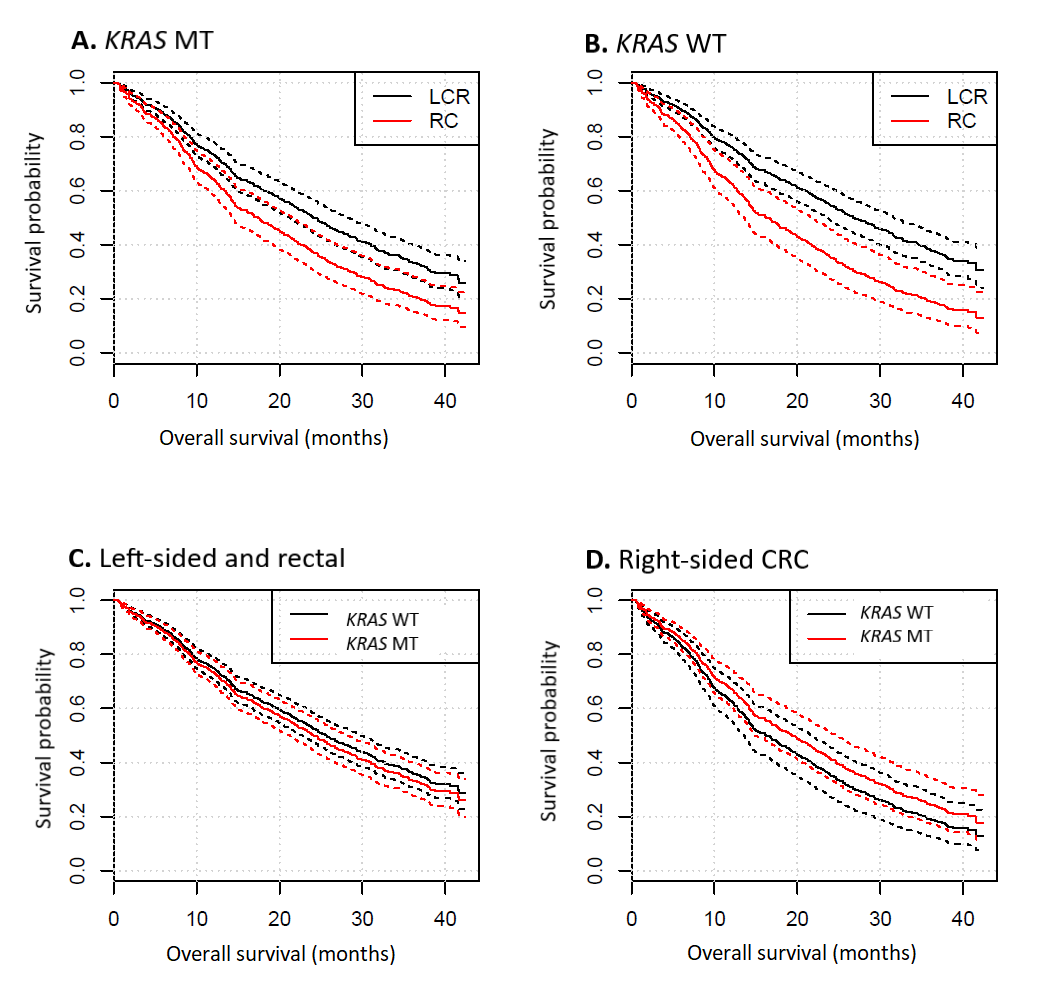

Supplement: oyad074_suppl_Supplementary_Materials [file oyad074_suppl_supplementary_materials.zip › oyad074_suppl_Supplementary_Figure_S2.tiff]

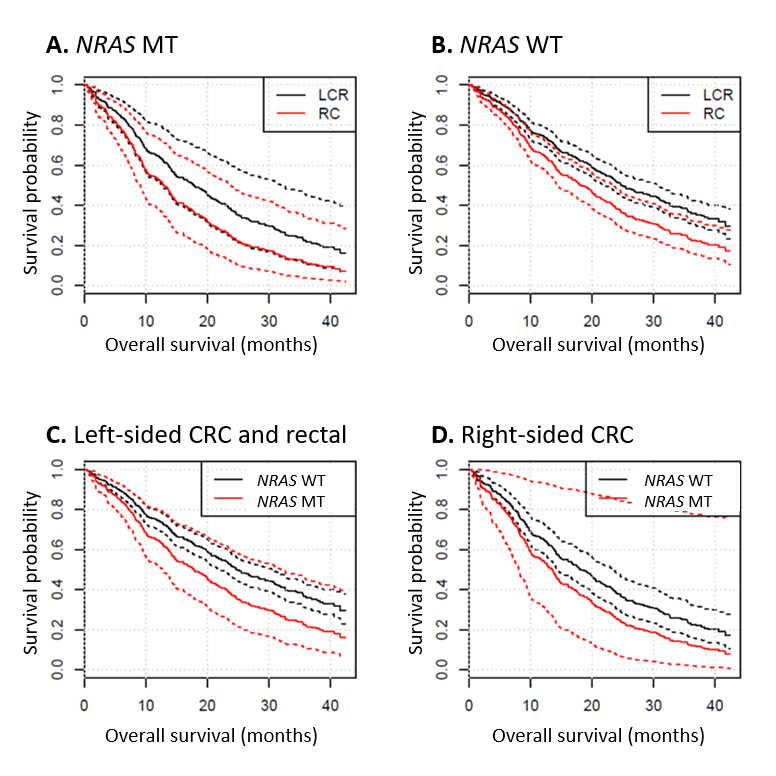

Supplement: oyad074_suppl_Supplementary_Materials [file oyad074_suppl_supplementary_materials.zip › oyad074_suppl_Supplementary_Figure_S3.tiff]

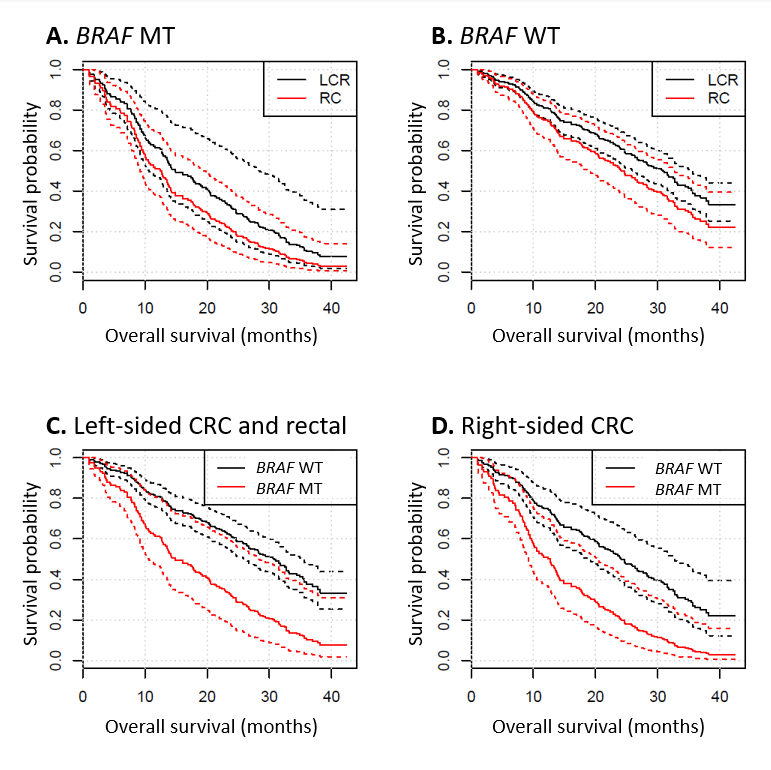

Supplement: oyad074_suppl_Supplementary_Materials [file oyad074_suppl_supplementary_materials.zip › oyad074_suppl_Supplementary_Figure_S4.tiff]

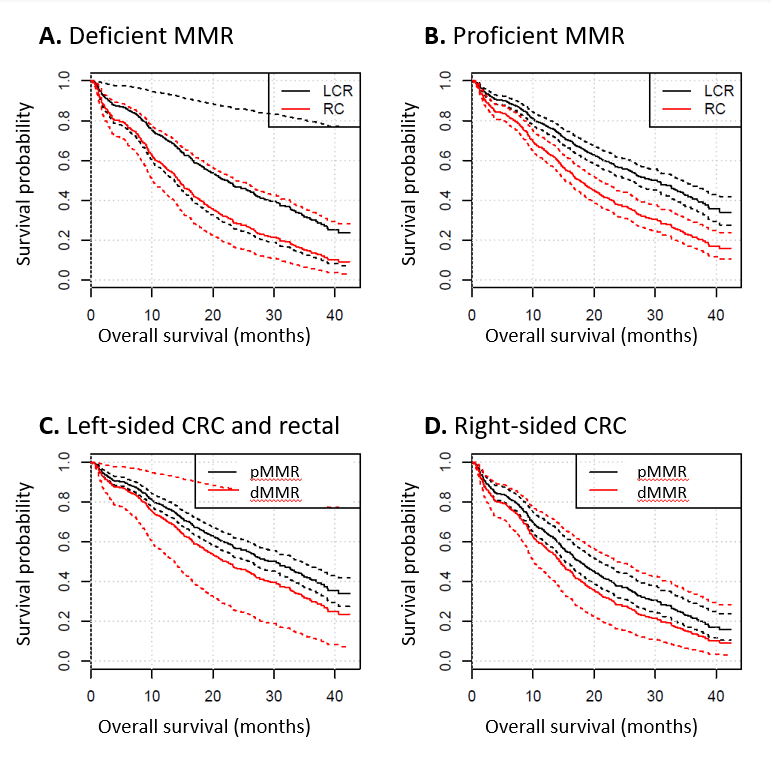

Supplement: oyad074_suppl_Supplementary_Materials [file oyad074_suppl_supplementary_materials.zip › oyad074_suppl_Supplementary_Figure_S5.tiff]

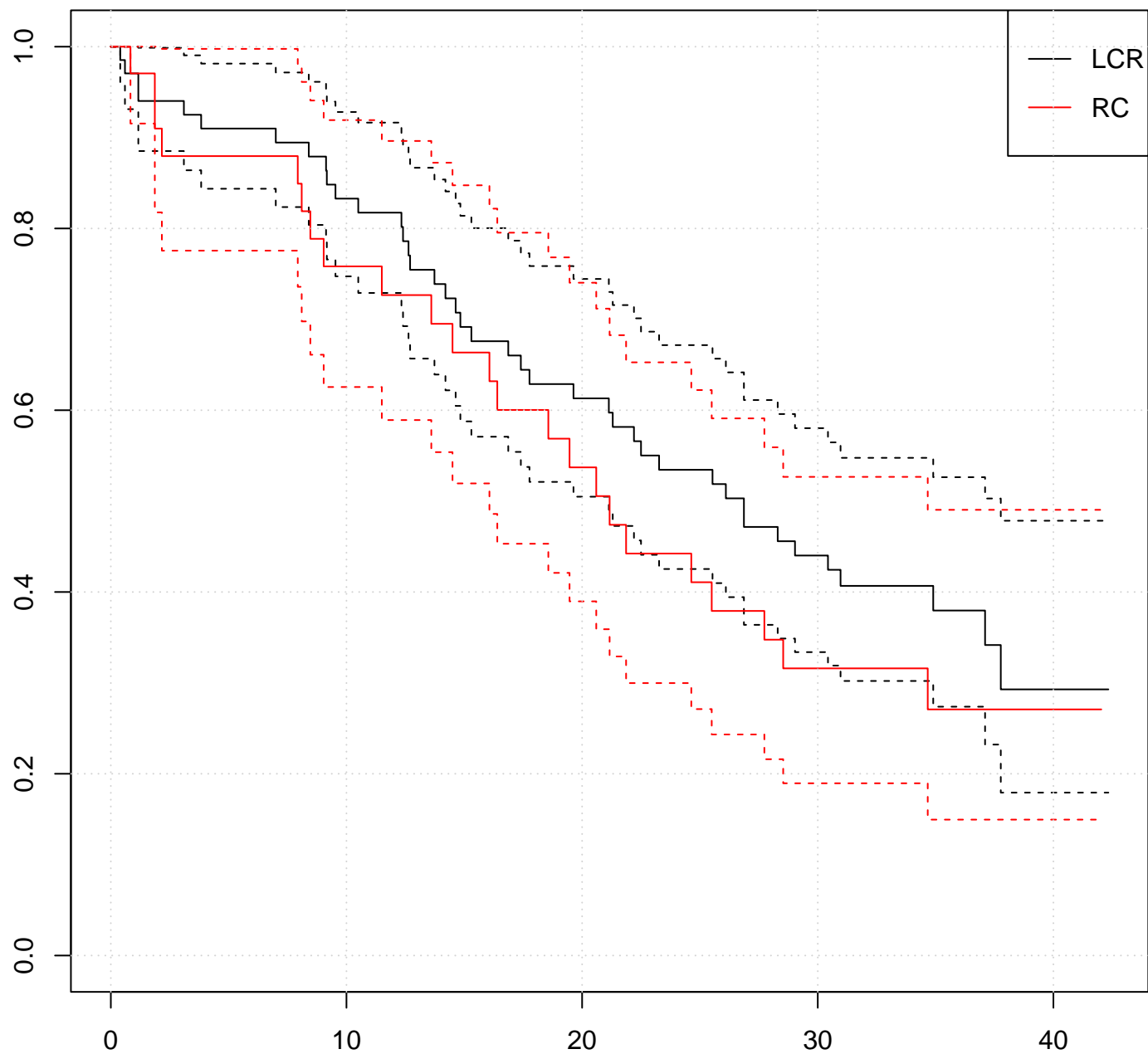

Supplement: oyad074_suppl_Supplementary_Materials [file oyad074_suppl_supplementary_materials.zip › oyad074_suppl_Supplementary_Figure_S6.pdf]

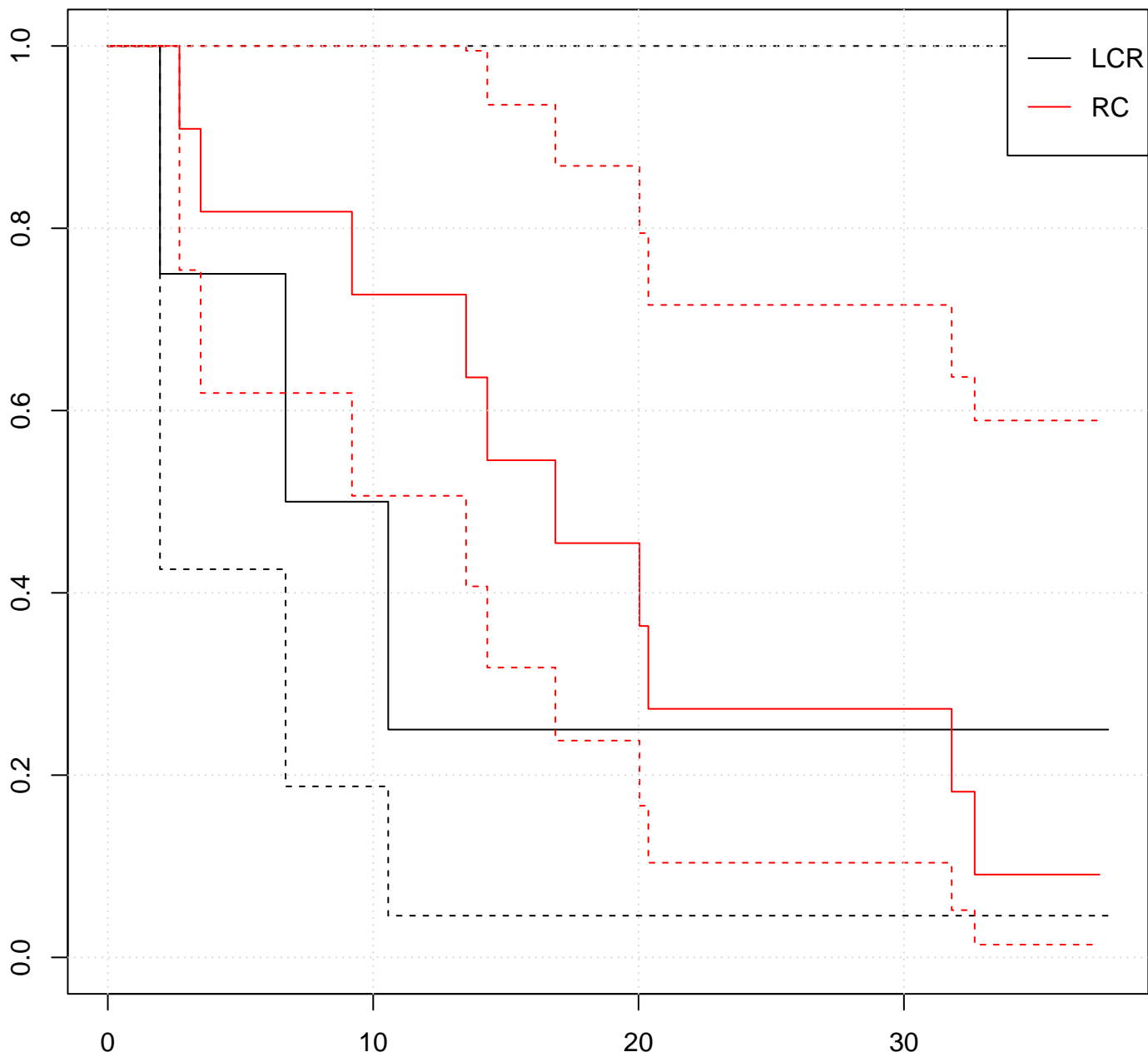

Supplement: oyad074_suppl_Supplementary_Materials [file oyad074_suppl_supplementary_materials.zip › oyad074_suppl_Supplementary_Figure_S7.pdf]

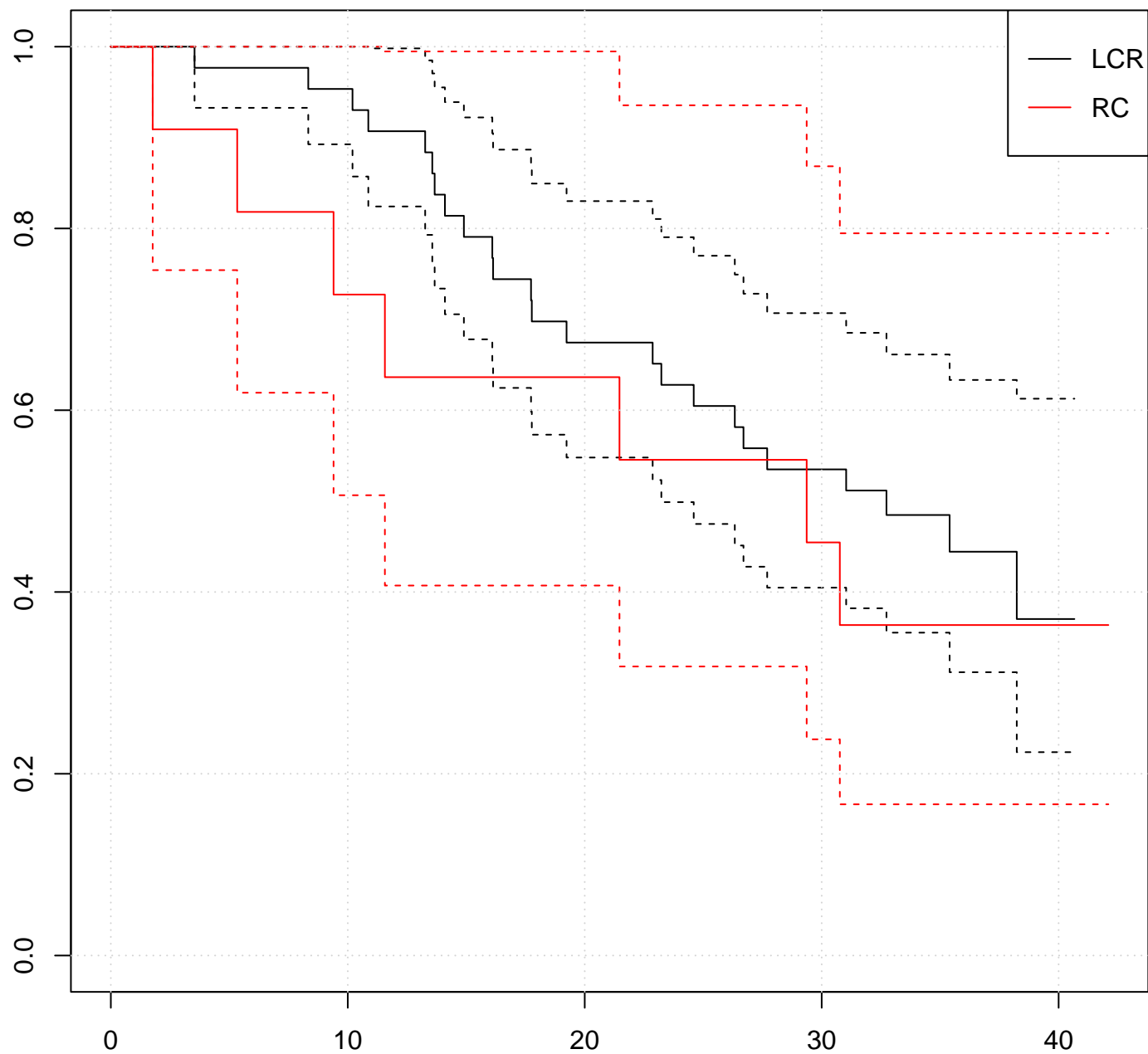

Supplement: oyad074_suppl_Supplementary_Materials [file oyad074_suppl_supplementary_materials.zip › oyad074_suppl_Supplementary_Figure_S8.pdf]

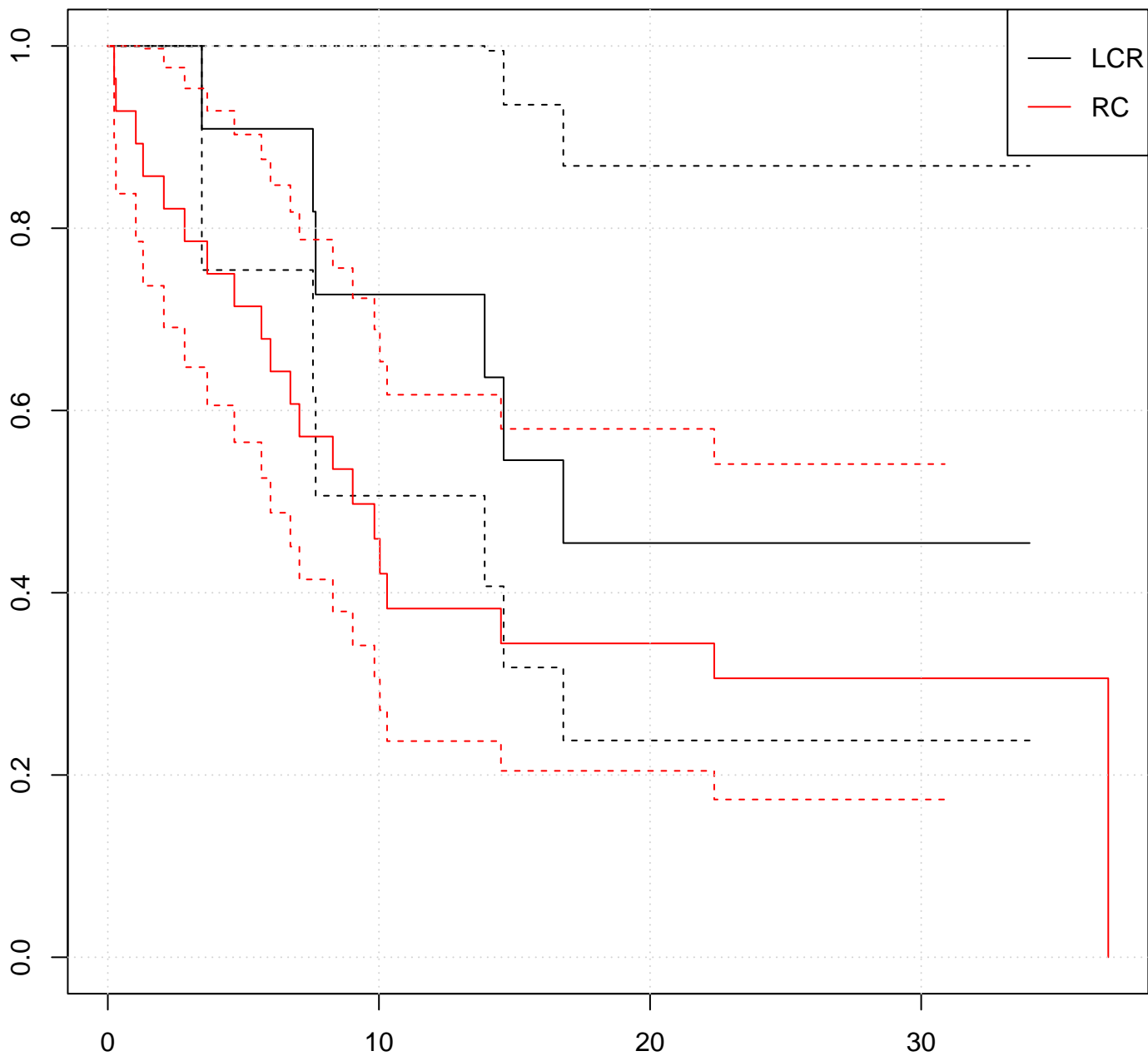

Supplement: oyad074_suppl_Supplementary_Materials [file oyad074_suppl_supplementary_materials.zip › oyad074_suppl_Supplementary_Figure_S9.pdf]

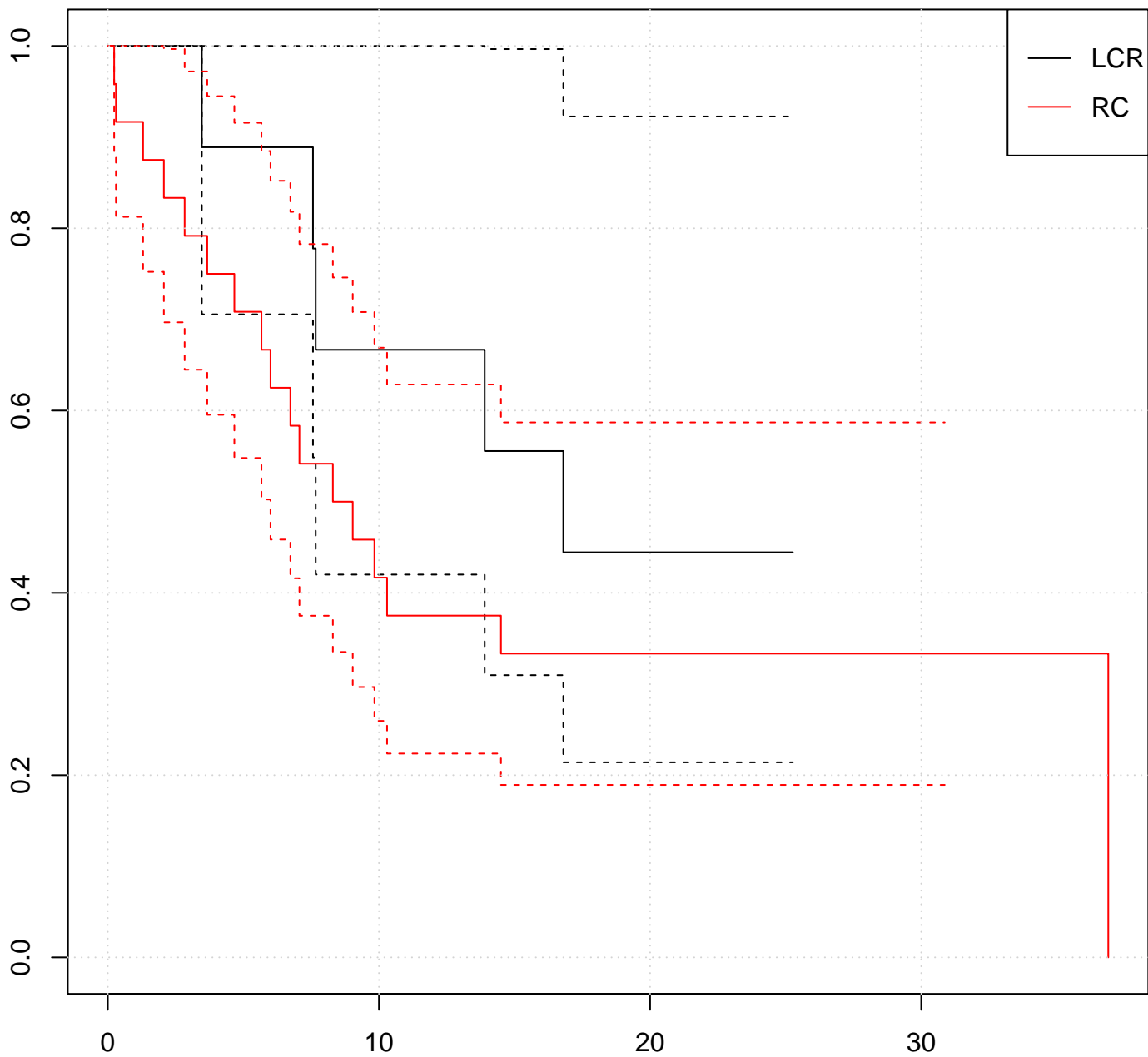

Supplement: oyad074_suppl_Supplementary_Materials [file oyad074_suppl_supplementary_materials.zip › oyad074_suppl_Supplementary_Figure_S10.pdf]
